# Cluster Analysis of Seismicity in the Eastern Gulf of Corinth Based on a Waveform Template Matching Catalog

**DOI:** 10.3390/s23062923

**Published:** 2023-03-08

**Authors:** Vasilis Kapetanidis, Georgios Michas, Ioannis Spingos, George Kaviris, Filippos Vallianatos

**Affiliations:** 1 Section of Geophysics—Geothermics, Department of Geology and Geoenvironment, School of Sciences, National and Kapodistrian University of Athens, Panepistimiopolis Zografou, 15784 Athens, Greece; 2 Institute of Physics of Earth’s Interior and Geohazards, UNESCO Chair on Solid Earth Physics and Geohazards Risk Reduction, Hellenic Mediterranean University Research Center, 71410 Heraklion, Greece

**Keywords:** Greece, Corinth, repeating earthquakes, template matching, seismicity migration, frequency–magnitude distribution, *b*-values, inter-event times

## Abstract

The Corinth Rift, in Central Greece, is one of the most seismically active areas in Europe. In the eastern part of the Gulf of Corinth, which has been the site of numerous large and destructive earthquakes in both historic and modern times, a pronounced earthquake swarm occurred in 2020–2021 at the Perachora peninsula. Herein, we present an in-depth analysis of this sequence, employing a high-resolution relocated earthquake catalog, further enhanced by the application of a multi-channel template matching technique, producing additional detections of over 7600 events between January 2020 and June 2021. Single-station template matching enriches the original catalog thirty-fold, providing origin times and magnitudes for over 24,000 events. We explore the variable levels of spatial and temporal resolution in the catalogs of different completeness magnitudes and also of variable location uncertainties. We characterize the frequency–magnitude distributions using the Gutenberg–Richter scaling relation and discuss possible *b*-value temporal variations that appear during the swarm and their implications for the stress levels in the area. The evolution of the swarm is further analyzed through spatiotemporal clustering methods, while the temporal properties of multiplet families indicate that short-lived seismic bursts, associated with the swarm, dominate the catalogs. Multiplet families present clustering effects at all time scales, suggesting triggering by aseismic factors, such as fluid diffusion, rather than constant stress loading, in accordance with the spatiotemporal migration patterns of seismicity.

## 1. Introduction

Earthquake behavior often manifests in patterns that are chiefly distinguished as mainshock–aftershock sequences and swarms. In the former case, a major earthquake leads the sequence, causing slippage of a relatively large fault surface that, depending on its magnitude, redistributes stress, which, in turn, triggers aftershocks to neighboring fault patches. Secondary sequences may then be triggered by major aftershocks, which are not expected to be much larger than approximately one order of magnitude less than the mainshock. There are, however, exceptions, with follow-up earthquakes having a magnitude comparable to that of the mainshock, as in several cases of earthquake “doublets”, which have been observed during the past decades in Greece [1,2,3]. In earthquake swarms, meanwhile, by definition, there is no single major event that could be characterized as a mainshock, leading the sequence. Very often, the larger events occur mid-sequence, rather than at the beginning [4], a characteristic that is exploited to distinguish between mainshock–aftershock sequences and swarms based on the skewness and kurtosis of their seismic moment release history [5]. Furthermore, swarms usually evolve in multiple stages, with bursts of earthquakes, usually concentrated in spatial clusters, either activating neighboring faults or re-activating the same fault patches [6,7]. The latter is related to the concept of repeating earthquakes, or multiplets, i.e., events with similar earthquake source parameters, producing nearly identical waveform recordings [8]. The spatiotemporal evolution of earthquake swarms usually exhibits migration patterns, with seismicity gradually spreading radially, or even unilaterally, from an origin through the fault network [9].

Triggering of seismicity can be enabled by Coulomb stress transfer due to the deformation caused by a major earthquake. Fault slip can also be facilitated by an increase in pore pressure [10]. The migration of seismicity during earthquake swarms can often be described as a spreading triggering front due to pore-pressure diffusion caused by high-pressurized pore fluids [11]. Earthquake swarms have been known to occur in areas related to active volcanism [12], involving fluids of magmatic origin or hydrothermal processes [13]. However, several studies in the Greek region have shown that swarms also occur in tectonic settings, with observed migration of seismicity implying interaction with fluids of mantelic or meteoric origin [14,15], or with the contribution of other aseismic factors, such as creeping [16]. 

To study the evolution of an earthquake sequence in detail, data from seismological stations at local epicentral distances must be collected. The utility of local networks is twofold: (1) seismic phase arrival times from stations near the epicentral area can help to constrain the hypocentral depths and define the geometries of the activated structures, and (2) an adequate number of local stations can greatly improve the detectability of low-magnitude earthquakes, whose signal-to-noise ratio is too low to be detected at longer distances. Mainshock–aftershock sequences, especially those involving large-magnitude events, usually trigger public interest, which, in turn, motivates seismological institutes to quickly establish temporary local networks in the affected area for closer monitoring of seismicity. The issue with earthquake swarms is that it is often not clear when a seismic excitation in a specific area, in terms of an increase in the seismicity rate, is going to last long enough [6], or evolve into a more hazardous event, such as a major earthquake [17,18], to merit the effort of deploying a temporary local network. As a result, most earthquake swarms usually go unnoticed or under-reported. 

**Figure 1:**
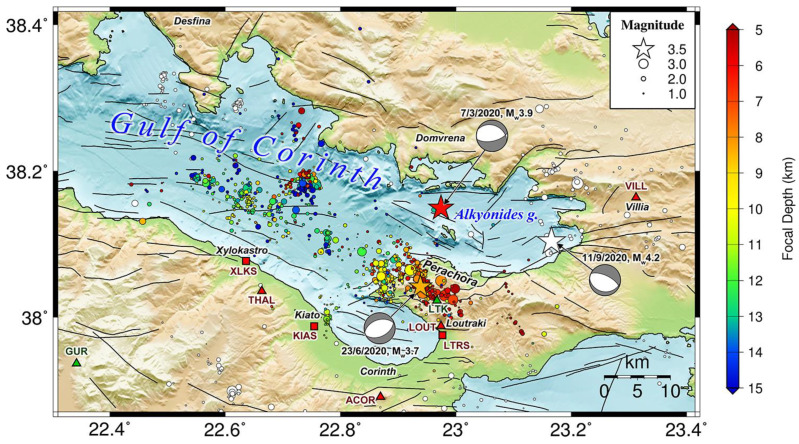
Area of study in the EGoC. Relocated seismicity for the period January 2020–June 2021, from the manual catalog of this study (CAT1), is presented with colors corresponding to the focal depth. Epicenters in the broader area with a white color are from the same period but not included in the catalog of this study. Events with magnitude *M* ≥ 3.5 are drawn with stars. Seismological (triangles) and accelerometric (squares) stations, belonging to HA ([46]; red) and HP ([47]; green) networks of the HUSN, which were operational during the study period, are also displayed on the map. Focal mechanisms of major events (*M_w_* ≥ 3.7) are from the databases of the NKUA-SL, GI-NOA, and ISC (see also [29]). Fault lines are from the NOAFaults v4.0 database [48,49].

In Greece, the implementation of the regional Hellenic Unified Seismic Network (HUSN), in 2008, has greatly improved real-time earthquake monitoring, both in terms of earthquake detectability, by lowering the magnitude of completeness, and reducing the location uncertainties by decreasing the azimuthal gap. The Seismological Laboratory of the National and Kapodistrian University of Athens (NKUA-SL) operates stations mainly distributed in Central Greece, where a major tectonic feature is the Corinth Rift, one of the most seismically active areas in Europe, dominated by ~E–W trending normal faults. The NKUA-SL first installed a permanent seismological network, the Cornet network, in 1995 in the Eastern Gulf of Corinth (EGoC) [19,20,21,22]. The Western Gulf of Corinth (WGoC) has been closely monitored since 2000 by the local Corinth Rift Laboratory network (CRL-net; [23]), with thousands of events detected and located every year. The EGoC, meanwhile (Figure 1), is covered by fewer local instruments, with a significant gap on its northern side and, unavoidably, to the west. Unlike the WGoC, where microseismic activity is continuously observed [7] and seismic swarms occur quite often [24,25], the EGoC area presents sporadic small earthquake clusters [26], usually offshore [27]. However, it has also hosted strong earthquakes in the past, with the more recent ones being a sequence of three *M* > 6 earthquakes in February–March 1981, which caused severe damage to the broader area and Athens ([28]).

Here, we revisit the earthquake swarm that occurred at the Perachora peninsula in the EGoC in 2020–2021. This seismic activity has been of particular interest as the epicentral area is located ~70 km west of Athens, the capital of Greece. The Perachora seismic swarm was initially investigated by [29], who presented its spatiotemporal characteristics through an analysis of a catalog of events that were detected, manually processed by routine analysis and relocated using the double-difference method. The main features of the sequence, such as its spatial properties, the occurrence of major events, and a general sense of spatiotemporal migration, could be determined from the data. However, a more detailed investigation requires a larger dataset, with a lower magnitude of completeness. This will enable the following:

- The possibility to examine potential temporal changes in the *b*-value of the Gutenberg–Richter scaling relation related to changes in the level of stress;
- The possibility to examine clustering effects of statistical properties of different multiplet families;
- The possibility to reveal changes in the seismicity rate unrelated to the occurrence of major events, hinting at possible triggering by external forcing (i.e., pressurized fluids or aseismic slip);
- The possibility to gain a more comprehensive view of the evolution of the swarm, e.g., by revealing earlier activation of certain areas or persistence of seismicity for longer than was previously known from routine analysis of data.

Concerning the latter, one such case is a major *M_w_* = 3.9 event that occurred on 7 March 2020, with only one aftershock associated with it in the routine catalog. An interesting question to be answered is whether there were more aftershocks or even foreshocks for this event. To that end, multiplet cluster analysis with an enhanced catalog can be used to investigate the evolution of the swarm at a localized level of seismic activity.

We examine data from local seismological stations to enhance the earthquake catalog with a large number of smaller events, detected through a matched filter technique [30]. Earthquake swarms, which are usually driven by aseismic factors such as fluids or slow slip, often exhibit repeated slip on certain small asperities. These spatially clustered events share similar source parameters, i.e., hypocenter and focal mechanism. As a result, such event groups, i.e., repeating earthquakes, or multiplets, produce similar waveform recordings. The matched filter method uses template waveforms of well-recorded and manually located events to scan the continuous recordings for other events with similar waveforms, which have not been included in the initial catalog. This method provides a plethora of parametric data, including origin information, P- and S-wave arrival times, and relative magnitude. 

The matched filter or template matching method has the potential to increase the number of available data by tenfold [31,32,33] or even more [34,35]. This technique has found many applications in case studies in the recent literature as it provides an automated way to drastically increase the volume of a dataset by detecting a large number of smaller events, previously unreported by routine analysis. Herath et al. [36] applied template matching, combined with a single-station travel time back-projection method, to detect an induced seismic swarm at the Victoria Reservoir in Sri Lanka, with events of magnitudes down to *M_w_* = −1.3. In another study, Chmiel et al. [37] combined the classic STA/LTA method [38] with template matching to detect 91 events using 23 templates. These events of magnitudes between −0.5 and 2.0 were triggered by hydrological loading or increase in the resulting in situ underground pore-pressure at the Maritime Alps, southern France, after the area was hit by Storm Alex in October 2020. Minetto et al. [39] applied a single-station matched filter to detect 79,503 events using 1330 templates in the area of Maurienne Valley in the western French Alps, during a swarm that occurred between August 2017 and March 2019. This enabled the authors to study the spatiotemporal migration of the sequence, the geometry of the activated faults, and the variation of the *b*-value of the Gutenberg–Richter relation, as well as gain a better understanding of the underlying physical mechanisms. Another example of single-station template matching is the case study of Stabile et al. [40], who detected 196 fluid-injection-induced seismic events of magnitudes down to −1.2 using eight templates in the area of the High Agri Valley, southern Italy, enhancing the spatiotemporal distribution of the swarm and correlating the seismicity with operational parameters such as injected volumes and pressures.

It is noted, however, that the enhanced catalog generated by template matching is usually of lower quality in terms of the involved uncertainties as the vast majority of the newly detected events will be below the magnitude of completeness of the initial catalog. This means that they comprise small-magnitude events, with low to very low signal-to-noise ratios, even at local stations, which is the primary reason why they were not detected by routine analysis in the first place. A correlation detector can, in some cases, discover events hidden in the ambient noise, albeit with a low correlation coefficient [41]. However, decreasing the correlation threshold to a low value can also lead to an increased number of false detections. It is also noted that the addition of a large number of small events to a catalog has a negligible effect on the cumulative seismic moment, as the larger events are dominant. As such, the application of template matching is not expected to affect the distinction between a mainshock–aftershock and a swarm pattern. 

Herein, we investigate modifications or improvements to the statistical parameters of the 2020–2021 seismic swarm in the Perachora peninsula that can be achieved through the application of the matched filter method. For this purpose, we compare the initial relocated catalog of [29] with two enhanced catalogs: one with additional events acquired through a multi-channel matched filter, single-event location, and double-difference relocation, and another produced by single-channel full-waveform template matching on a single local station (LTK; Figure 1) in the vicinity of the major spatial clusters of the sequence. The first catalog has fewer detections, but is considered of better quality, as the events were detected by more than one stations and are more reliable; the second catalog has more detections, as only the vertical component of one reference station was used, but has increased probability of false detections. Despite the limitations or biases in these catalogs, the matched filter method manages to increase the number of events by tenfold through multi-channel detection and by thirty-fold through single-channel template matching. 

In the present study, we utilize the enhanced catalogs to investigate the statistical properties of microseismicity in the area, with a particular focus on the Perachora swarm. Initially, we explore the magnitude of completeness of each catalog and the frequency–magnitude distribution (FMD), which is an essential part of any seismic hazard analysis. The FMD is commonly described with the Gutenberg–Richter scaling relation [42], yielding the value of the seismic parameter *b*, which expresses the relative number of smaller to larger magnitude events in a seismic region. The plethora of detected events in the enhanced catalogs further allows us to investigate possible temporal *b*-value variations during the swarm that might be associated with local stress changes [43,44,45]. We further explore the evolution of the swarm through spatiotemporal clustering methods, and possible migration patterns that might be associated with pore fluid pressure diffusion, which is most likely the triggering mechanism of the swarm, according to [29]. In addition, we investigate the temporal properties of multiplet families through statistics concerning the inter-event times (or waiting times) between the successive events and discuss the clustering effects that emerge in the temporal evolution of seismicity.

## 2. Materials and Methods

### 2.1. Template Matching Catalogs

This study builds on data and results that were first presented by [29] and later used by [50]. The catalog includes absolute locations for 828 events, located via an optimal velocity model for the study area, of which 788 events were relocated using the double-difference method (HypoDD; [51]) to minimize relative location uncertainties. These events were all automatically detected and then manually located via routine analysis at the NKUA-SL and the Geodynamics Institute of the National Observatory of Athens (GI-NOA). Waveform data from the stations of the Hellenic Unified Seismic Network (HUSN), used in this analysis, were obtained through the EIDA node [52], hosted at the GI-NOA. 

Routine catalogs ensure adequate location uncertainties based on P- and S-wave arrival time data from several local or regional stations. Catalogs are complete down to a minimum magnitude of completeness, *M_c_*, which, in this case, was found at *M_c_* = 1.6 [29]. In order to lower the magnitude of completeness and enhance the catalog with a large number of smaller events, different methodologies can be applied. Deep learning techniques can be used to automatically pick seismic phase arrivals, recognize them as P- or S-waves, and associate them with specific earthquake events [53]. However, a minimum number of stations is required to determine an absolute hypocentral location. This becomes more difficult at smaller magnitudes as the signal-to-noise ratio can become too low at stations that are not near the epicenters. Meanwhile, methods that involve the application of waveform cross-correlation for template matching are capable of detecting earthquake signals with very low signal-to-noise ratios [41] and associating them with known origins. However, these methods rely on waveform similarity, which means they cannot be used to detect events that are dissimilar to any of the templates in a database. Furthermore, there is still a requirement for a minimum of 3–4 stations in order to acquire absolute hypocentral solutions with a single-event location algorithm. Template matching on a single station in the vicinity of the epicentral area can maximize the number of repeating earthquakes that are detected, providing a great resolution of event occurrences in the time domain; however, the hypocentral locations of detected events will need to be fixed to the same focus as that of the template that was used for their detection.

In this study, we apply the template matching method EQcorrscan [30] in order to enhance the catalog of [29] with a large number of repeating earthquakes. This algorithm takes into account an initial database of template events with well-established solutions. The main steps of the procedure are summarized below:

(1) Preparation of the templates dataset (tribe): waveform processing (down-sampling, filtering, P- and S-wave cropping at all available channels), parametric information sourcing (hypocenter, origin time, P- and S-wave travel times, magnitude) from the manual catalog;
(2) Construction of a database of continuous recordings to be scanned for detections;
(3) Application of the matched filter to make detections based on the template waveforms (families), performed on a day-by-day basis;
(4) Merging of families constructed with the same template on different days into a single “party” (set of families);
(5) Declustering of the party, removing duplicate detections by keeping those with the highest correlation coefficient;
(6) Determination of relative magnitude for detections with an adequate correlation coefficient and signal-to-noise ratio;
(7) Finetuning of the time lag to provide detections with more accurate arrival times for the P- and S-waves (multi-channel matched filter only).

Herein, we used the catalog of [29] for the templates, including a few additional events in the eastern part of the Perachora peninsula, amounting to a total of 879 events, of which 808 were relocated using the double-difference procedure described by [29]. This catalog is hereon termed “CAT1”. The *M_L_* magnitude was available for the templates; as described later on, that was used for the estimation of the relative magnitude for the detections. In the terminology adopted by EQcorrscan, the complete set of templates constitutes a “tribe”. Each template is used to scan the continuous recordings for matching waveforms, called “detections”. The detections made with a certain template comprise a “family”, and a set of families constitutes a “party”. The data were processed on a day-by-day basis using all templates of a tribe, so a party of families was first created for each day, keeping only those templates that provided at least one detection. During the post-processing of the results, families of the same template in parties formed on different days were merged into a single family, containing detections of all days. All families were finally merged into a single party for the whole period.

The template waveforms were prepared by down-sampling from 100 to 50 samples/sec to reduce the processing time, detrending, and applying a bandpass Butterworth filter between 2 and 15 Hz to lessen long-period distortions and remove high-frequency noise from the signals. A limited number of stations, located near the shores of the Eastern Gulf of Corinth (EGoC), were used for their waveform data. Templates were created by cropping the waveforms of these stations’ recordings for windows of P- and S-waves with available arrival times. EQcorrscan requires one type of wave for each station’s channel, so P-wave windows are trimmed on the vertical component and S-waves on the two horizontal components. Each template includes all available channels, with P- and S-waves of each station positioned at an appropriate time lag, corresponding to the difference in the travel times between different seismic phases and stations. The template also includes parametric information about its corresponding event, i.e., the hypocentral coordinates, origin time, travel times of the P- and S-waves at each station, and the *M_L_* magnitude. The specific parameters used for the preparation and filtering of the template waveforms are also stored, and the same processing is later applied to the continuous recordings before the application of the template matching.

**Figure 2:**
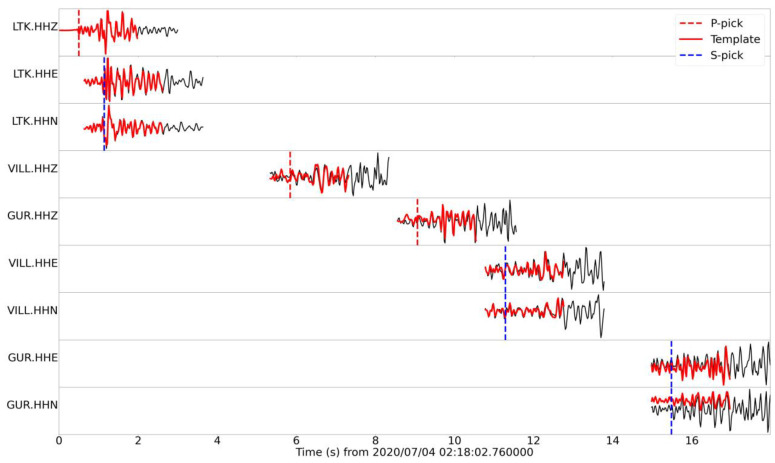
Example of the application of the EQcorrscan code for the detection of similar earthquakes with the template matching technique. The template waveforms are presented in red, while the part of the continuous record with detected signals is represented in black. The arrival times of P- and S-waves are depicted by vertical dashed red and blue lines, respectively. The different channels are divided by horizontal black lines and sorted by ascending arrival time of the respective seismic phase.

As the chunks of continuous recordings are scanned during the matched filter earthquake detection procedure, waveform similarity is measured in terms of the correlation coefficient for each available channel, at their respective relative time lags, depending on the travel time differences between their P- and S-waves (Figure 2). A metric is required to measure the average degree of similarity. When an adequate number of channels is available, the median absolute deviation (MAD) is usually preferred, which functions as a metric for the average distance between the data and the median, acting as a measure of similarity. 

Here, the matched filter procedure was applied in two different ways. Initially, a tribe of templates was formed by using only the vertical channel of station LTK, which is the closest to the main clusters of the Perachora swarm and the most complete in terms of continuous waveform data for the whole period under study. Template matching was performed by employing the whole signal waveform, including both P- and S-waves, and using the absolute correlation metric with a threshold value of 0.6, which initially produced over 74,000 signal detections. It is noted, however, that these are non-unique as many could be triggered by different templates with similar waveforms. Detections of events corresponding to the templates are also possible via either autocorrelation or by having a similar earthquake in the tribe that detected them. As such, the results have to be post-processed so that duplicate events are removed. For detections that have been found by different templates, the one with the higher similarity value is retained. The catalog formed via single-channel detections on the vertical component of station LTK will be hereon labeled “CAT2”.

Afterward, multi-channel matched filtering was performed using the tribe of templates created from the selection of the available local stations, with separate windows for P- and S-waves trimmed at the vertical and horizontal components, respectively. The MAD metric was utilized as a measure of similarity, with a threshold of 8, i.e., requiring that the signal was eight times above the noise level to register a valid detection, selected after some trial-and-error tests. The trigger times of the over 74,000 detections, originally determined only with the reference station LTK, were used as a guide to optimize the procedure and avoid re-processing the whole length of the continuous multi-channel records. As a further step, after the initial scan for the multi-channel matched filter, the new detections were re-processed by allowing the template waveforms of each channel to be temporally shifted by ±0.5 s about the average detection time. This enabled the finetuning of the P- and S-wave arrival times at each station, to account for the likely minor offset between the hypocenter of the template and each detection. The catalog formed from the multi-channel detections was labeled “CAT3”, which initially included over 23,000 events. The detections of CAT3 were evaluated by performing single-event location with the VELEST code [54], employing the velocity model of [29]. This resulted in a total of over 10,759 detected and resolved events after the exclusion of duplicates and templates.

For both datasets of detections (CAT2 and CAT3), the relative magnitude was also measured. This was determined according to the formula of Equation (1) (based on [55]):

(1) $$\Delta m=\log\frac{std\left(tr_{2}\right)}{std\left(tr_{1}\right)}+\log\frac{\left(1+1/snr_{2}^{2}\right)}{\left(1+1/snr_{1}^{2}\right)}\times CC,$$

where Δ*m* is the magnitude difference between template and detection, measured for one channel, with the amplitudes of their traces symbolized as “*tr*_1_” and “*tr*_2_”, respectively, *std* being their standard deviation, *snr*_1_ and *snr*_2_ the respective signal-to-noise ratios, and *CC* the correlation coefficient, used as a weight. As described by [55], the relative size of a detection compared to a template is based on the amplitude scaling factor “*a*” of [56], which is essentially the unnormalized cross-correlation coefficient between the two waveforms. The amplitude ratio (scaling factor) “*a*” is expressed by the first term on the right side of Equation (1) as the ratio between the standard deviations of the trace amplitudes. The relative magnitude Δ*m* is then equal to log*a*. The time series can be concatenated to include all the available channels and inverted for a single-amplitude scaling factor. The second term in Equation (1) is used to take into account the biases introduced to the relative magnitude calculation by the correlation coefficient and the signal-tο-noise ratio [55].

By subtracting Δ*m* from the *M_L_* magnitude of the template, we acquire an estimation of the magnitude for each detection. As a minimum correlation value and signal-to-noise ratio are required for the determination of the relative magnitude, Δ*m* can only be determined for a subset of detected events. This yielded relative magnitudes for 23,523 detections (after the exclusion of duplicates) in CAT2 and 7648 detections in CAT3.

### 2.2. Clustering and Relocation

In the work of [29], as a part of the relocation procedure, the waveforms of the events in the manual catalog (CAT1) were cross-correlated, forming a matrix with the cross-correlation maxima between all combinations of event pairs. Agglomerative clustering followed, with nearest-neighbor linkage, and multiplets were formed with a correlation threshold of 0.64. One of the clustering schemes we used in the present study is based on the results for the abovementioned multiplets: the detections in CAT2 and CAT3 are registered to the same multiplet as the one to which their associated template belongs. Each multiplet is assigned an ID. The IDs, to be more useful, are sorted in ascending order according to the origin time of the first event in each cluster, including detections that are incorporated in the original multiplets. Furthermore, events of CAT1 that were initially not associated with a multiplet, or even a doublet (multiplet of two events), are also assigned a unique cluster ID. If such a template event has a family of detections in CAT2 or CAT3, these will form a regular multiplet.

Nearest-neighbor linkage can be useful for small numbers of relatively incomplete data as it tends to create broad clusters of the more similar events, but with relatively weak consistency. The weakness of this method is that when the matched-filter detections are incorporated into these clusters, some may become too large compared to the others, and dissimilarities between subclusters within a large multiplet can be significant. To account for this issue, in addition to the nearest-neighbor clustering performed by [29], here, we also performed farthest-neighbor linkage to the events of CAT1, forming a large number of smaller multiplets, starting at a low threshold of 0.5. This ensured that no event pair within a multiplet had a correlation maximum of less than 0.5 (nearest neighbor linkage does not prevent this problem). The detections incorporated into the set of multiplets formed with farthest-neighbor linkage will likely not comply with this rule as they are only strictly similar to their associated template. Furthermore, the lower signal-to-noise ratio, due to their smaller magnitude, reduces their correlation values. As in the previous case, for each clustering scheme applied to CAT2 and CAT3, the multiplet IDs (MID) were sorted by ascending order of the origin time of the first event, and single templates (not belonging to one of the initially formed multiplets) were also assigned a unique MID. Clustering with farthest-neighbor linkage was performed for threshold values of 0.6, 0.7, 0.8, and 0.9.

**Figure 3:**
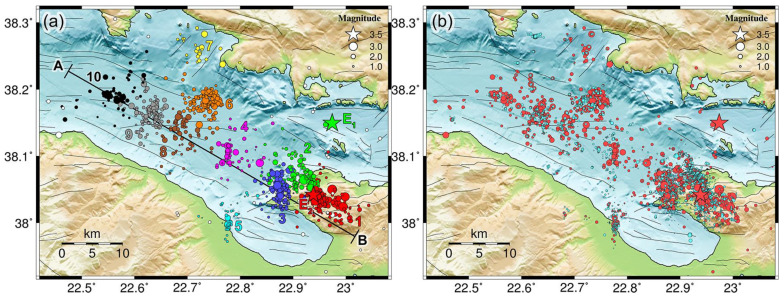
Map of seismicity in the Eastern Gulf of Corinth between January 2020 and June 2021. (**a**) Catalog of routinely analyzed relocated events (CAT1), also used as templates, with different colors and numbers representing 10 different spatial groups. White color is used for events not incorporated into a spatial group. E_1_ denotes a major isolated event (green star) with *M_w_* = 3.9 that occurred on 7 March 2020 in the Alkyonides gulf; E_2_ is the major *M_w_* = 3.7 event of 23 June 2020 at Perachora (red star). (**b**) Catalog of templates (red) and relocated detections (cyan) from CAT3. Only detections with a calculated relative magnitude are displayed.

Lastly, in the work of [29], the catalog was separated into 10 spatial groups. These were formed using Ward’s linkage on the matrix of 3D inter-event distances relocated using the double-difference method. In the present study, we adopted the same spatial groups and incorporate matched filter detections into them according to the group that was assigned to their associated template. The distribution of the 10 groups is presented in Figure 3a. Groups #1, #2, and #3 comprise the bulk of seismicity with epicenters on the Perachora peninsula, mainly activated between March and September 2020.

Next, we applied double-difference relocation to the detections of CAT3, making use of the larger spatial groups to break down the dataset into smaller, manageable parts. For that purpose, for events within each spatial group, we performed cross-correlation measurements for P- and S-waves between event pair combinations with available arrival times imposed by the matched filter method at common stations. Likewise, we used the travel times determined with the VELEST model of [29] as catalog input data for the double-difference method. We used the hypocenters of the associated templates as initial hypocenters for the detections. As the available measurements for each event were very few due to the lack of a dense local network in the area and the strong waveform similarity for a measurement to be made, the parameters for HypoDD were set at a bare minimum requirement for links between neighboring events. Catalog links mainly kept the different subclusters together, while cross-correlation data were primarily used for relocation. The drawback was that the relocated hypocenters of events with few data tended to align along artificial lines. This was especially the case for several events of small magnitude in the dense clusters below Perachora, which were detected mainly due to station LTK being in the epicentral area. Events of other spatial groups, further away from the peninsula, seemed to be less affected by this issue. Despite their low quality, the relocated solutions for 10,750 detections in CAT3 (7644 with a relative magnitude estimate) created a spatial scatter about their initially fixed location (Figure 3b) at the hypocenter of their associated template and provided an enhanced and slightly more realistic spatiotemporal distribution.

## 3. Results

### 3.1. Frequency–Magnitude Distribution and Catalog Completeness

**Figure 4:**
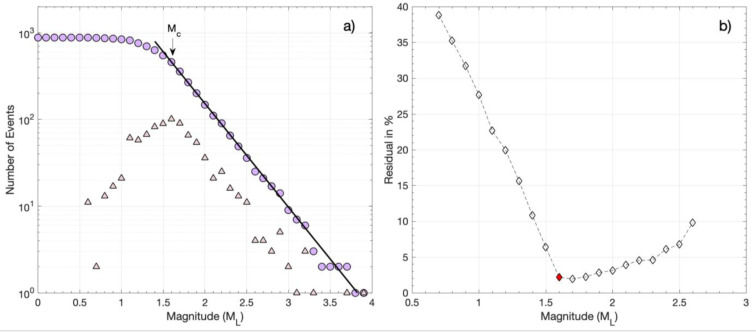
(**a**) Cumulative (filled circles) and discrete (filled triangles) frequency–magnitude distribution for CAT1. The solid line represents the Gutenberg–Richter (G–R) scaling relation for *a* = 4.57 and *b* = 1.20. (**b**) Residual plot between the observed frequency–magnitude distribution and the fit to the G–R relation as a function of the lower magnitude cut-off. The filled red symbol represents the *M_c_* of 1.6 for 95% residuals.

To estimate the magnitude of completeness (*M_c_*) for the three catalogs, i.e., CAT1, CAT2, and CAT3, we use the maximum curvature (MaxC) method and the goodness-of-fit (GoF) test [57]. The original catalog of [29], with a total of 828 events, had a magnitude of completeness *M_c_* = 1.6 (Figure 4). Herein, the catalog includes a few additional events (CAT1), forming a total of 879 events. The frequency–magnitude distribution (FMD) of CAT1 is shown in Figure 4a. The MaxC method for CAT1 provides an *M_c_* of 1.5, similar to the one provided by the GoF test for 90% residuals (Figure 4b). For 95% residuals, however, the GoF test provides an *M_c_* of 1.6 (Figure 4b), which is the magnitude threshold preferred for CAT1, as for *M_c_* = 1.6, the maximum likelihood solution [58] of the Gutenberg–Richter (G–R) scaling relation (i.e., log*N* = *a* − *bM*, where *N* is the number of earthquakes with magnitudes equal or greater than *M* and *a* and *b* are scaling parameters [42]) provides a better fit to the observed FMD (Figure 4a). The *a* and *b* parameters of the G–R relation and their associated uncertainties [59] for *M_c_* = 1.6 are *a* = 4.57 ± 0.15 and *b* = 1.20 ± 0.05.

**Figure 5:**
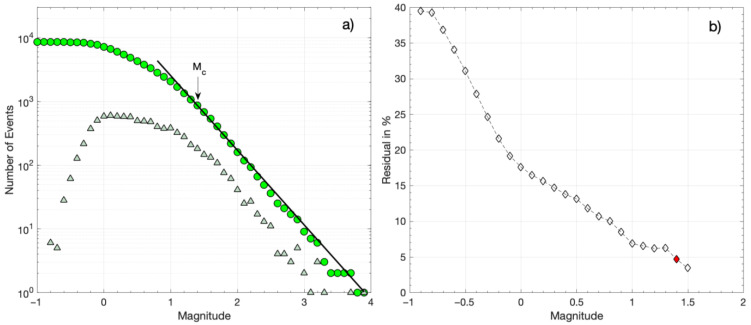
(**a**) Cumulative (filled circles) and discrete (filled triangles) frequency–magnitude distribution for CAT3. The solid line represents the G–R scaling relation for *a* = 4.58 and *b* = 1.17. (**b**) Residual plot between the observed frequency–magnitude distribution and the fit to the G–R relation as a function of the lower magnitude cut-off. The filled red symbol represents the *M_c_* of 1.4 for 95% residuals.

The catalog of multi-channel detections, CAT3, contains a total of 8527 events with a magnitude estimate (either *M_L_* for templates or relative magnitude for detections). The minimum magnitude of CAT3 is estimated at −0.8. The FMD for CAT3 is shown in Figure 5a. The number of events keeps increasing, down to magnitude 0.5. However, the absolute value of the slope between 0.5 and 1.4 appears to be lower than for magnitudes above 1.4. This could be caused by a real deficit of detected events in the catalog, i.e., events that could not be detected because they were dissimilar from all used templates, or not similar enough for an adequate number of channels. It could also be due to a real difference in the *b*-value for events of smaller magnitudes, indicating different scaling in the distribution of magnitudes, reflecting differences in the distribution of rupture lengths or areas. A simpler explanation is that there is a difference in the way that the magnitude is determined between the templates and the detections, so a bias in the measurements of relative magnitude is to be expected, which could be dependent on the correlation coefficient [55]. For the templates of CAT1, the *M_L_* magnitude is calculated, taking into account instrument correction and the peak amplitude of the waveforms. Meanwhile, for the detections of CAT3, a relative magnitude is estimated, considering the standard deviation ratio and signal-to-noise ratio between the traces of the templates and detections (Equation (1)). This type of magnitude will likely have different scaling from *M_L_*, which could explain the different slope in the FMD. Nevertheless, the MaxC method and the GoF test for 90% residuals (Figure 5b) provide an *M_c_* of 0.9 for CAT3. The *M_c_* is higher for 95% residuals, equal to 1.4 (Figure 5b). The latter value of *M_c_* = 1.4 is preferred as when setting the minimum magnitude to 1.4 for the maximum likelihood estimation of the *a* and *b* parameters, the G–R relation has a better fit to the observed distribution (Figure 5a). The *a* and *b* parameters are estimated as *a* = 4.58 ± 0.13 and *b* = 1.17 ± 0.04, which are almost identical to those calculated for CAT1. 

The CAT2 catalog contains a larger number of events, but its spatial distribution is not improved as the hypocenters of detections are fixed to those of the associated templates. There is a greater chance of false detections as only the vertical channel of a single reference station was used. This is usually the case when a template of a relatively low signal-to-noise ratio (e.g., from an event of low magnitude or relatively far from station LTK) happens to be similar to random noise signals. In that case, a large number of false detections can be produced. It is also possible for templates of a relatively higher magnitude and signal-to-noise ratio to match with signals of events (or impulsive local noise) that are not similar when their correlation coefficient is above the threshold. This is less likely to occur with multi-channel/multi-station detections, but to minimize its occurrence, it is required that an adequate number of stations near the epicenters be operational throughout the period of study. There is a trade-off between maximizing the number of detections and keeping the invalid triggers to a minimum. Setting a higher correlation threshold reduces the number of false detections, but may also remove real events with correct associations to templates.

**Figure 6:**
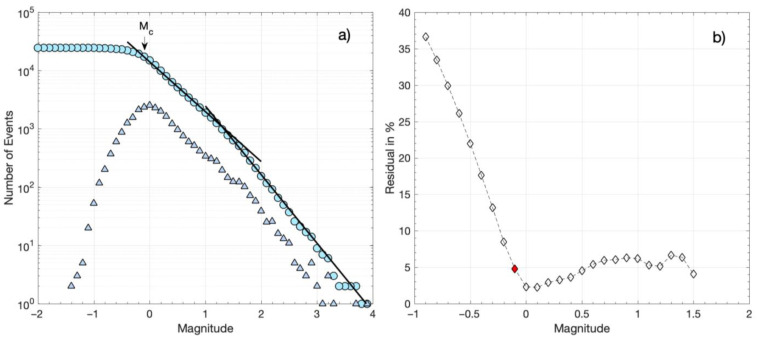
(**a**) Cumulative (filled circles) and discrete (filled triangles) frequency–magnitude distribution for CAT2. The two solid lines represent the G–R relation for the parameter values of *a* = 4.15 and *b* = 0.86 for a cut-off magnitude −0.1, and *a* = 4.57, and *b* = 1.18 for a cut-off magnitude of 1.5. (**b**) Residual plot between the observed frequency–magnitude distribution and the fit to the G–R relation as a function of the lower-magnitude cut-off. The filled red symbol represents the *M_c_* of −0.1 for 95% residuals.

Artifacts such as those described above were initially detected in CAT2. They became apparent due to a significant outlier in the FMD (many false detections reported the same magnitude) but also in the spatiotemporal distribution as multiple triggers at the same hypocenter throughout the whole study period were present with no apparent reason. Visual inspection of the suspected false detections confirmed the issue, and the detections of low correlation coefficient produced by such templates were removed from CAT2. The intervention improved the FMD shown in Figure 6a, with the lowest registered relative magnitude being −1.4. The *M_c_* of CAT2, as estimated with the MaxC method and the GoF test for 90% residuals, is −0.2, while for 95% residuals, the *M_c_* is −0.1 (Figure 6b). For this latter value, the parameters of the G–R relation are estimated as *a* = 4.15 ± 0.09 and *b* = 0.86 ± 0.01. However, as can be seen in Figure 6b, for these parameter values, there is a significant misfit of the G–R relation for magnitudes above ~1.4. A second G–R relation then appears for *M* > 1.4, defining a bimodal distribution with *a* = 4.57 ± 0.13 and *b* = 1.18 ± 0.04 for higher magnitudes (Figure 6a). These *a* and *b* values are similar to those for CAT1 and CAT3. The bimodal FMD of CAT2 may be an artifact, either due to a lack of detections for smaller-magnitude events or due to the determination of relative magnitudes mixed with *M_L_* magnitudes, as was discussed previously for CAT3. Alternatively, the bimodal FMD may be a true scaling property, where smaller-magnitude events follow the G–R relation with a lower *b*-value.

We further explored the FMD for the events that occurred on the Perachora peninsula, which hosted the earthquake swarm during 2020, the most prominent sequence during the studied period. We considered the events belonging to spatial groups #1, #2, and #3 with epicenters on the Perachora peninsula close to the station LTK that was used for the single-station detections of CAT2. These spatial groups, hosting the events of the swarm, are the most populous in the three catalogs. Specifically, ~40.5% of the events in CAT1, ~91.7% in CAT2, and ~71.9% in CAT3 belong to these three spatial groups. The percentages in the three catalogs are not proportional, verifying that the enhanced CAT3 and particularly CAT2 register more detections from template-matching belonging to spatial groups 1–3, close to the station LTK, than the other spatial groups, which are mainly located offshore (see Figure 3a).

**Figure 7:**
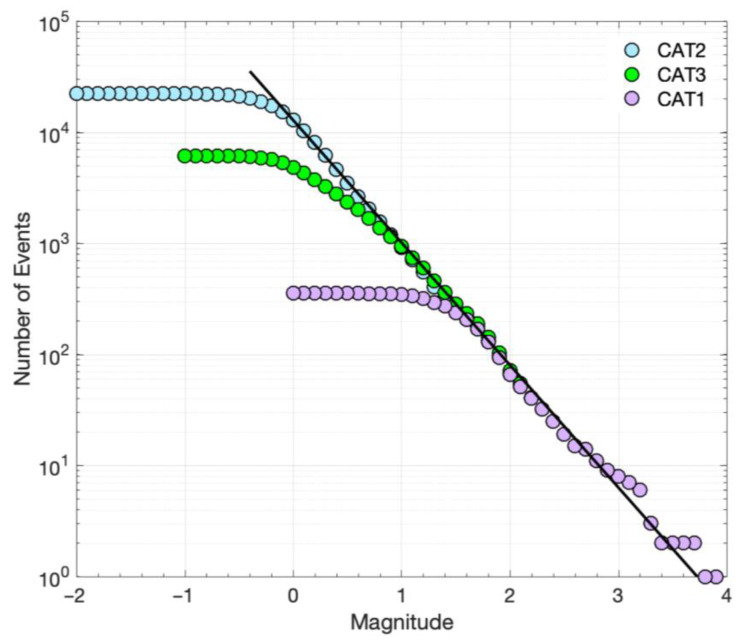
Cumulative frequency–magnitude distributions (filled circles) for the events in spatial groups 1–3 (main groups on Perachora peninsula) for the three catalogs (CAT1, CAT2, and CAT3). The solid line represents the G–R scaling relation for the parameter values *a* = 4.11 and *b* = 1.10.

The cumulative FMD values for spatial groups 1–3 in the three catalogs are shown in Figure 7. The MaxC and the GoF test for 90% residuals indicate an *M_c_* of 1.5 for CAT1, −0.1 for CAT2, and 0.1 for CAT3, while for 95% residuals, *M_c_* equals 1.6 for CAT1, 0.0 for CAT2, and 0.9 for CAT3 (Table 1). In Figure 7, we can observe that the three FMD values are similar for *M* ≥ *M_c_* (for 95% residuals), i.e., *M* ≥ *M_c_* = 1.6 for all three catalogs and *M* ≥ *M_c_* = 0.9 for CAT2 and CAT3. In addition, the FMD of CAT2 is continuous and linear down to *M* = *M_c_* = 0.0, showing a good fit with the G–R relation for *a* = 4.11 ± 0.11 and *b* = 1.10 ± 0.01 (Figure 7). The similarity between the three FMDs in spatial groups 1–3 indicates that template matching manages to reduce the *M_c_* from 1.6 in the original catalog (CAT1), to 0.9 in the multiple-station detection catalog (CAT3), and finally, to 0.0 in the single-station detection catalog (CAT2). The continuous FMD down to *M* = 0.0 for CAT2 further suggests that mixing different types of magnitudes in the distribution, i.e., relative magnitudes and *M_L_*, does not affect the FMD. The discontinuity that is then observed in the FMD of CAT3 for *M* < 0.9, or *M* < 1.4 in the entire catalog (Figure 5), most probably reflects underrepresentation of small-magnitude detections [60]. The same probably applies to the entire CAT2, resulting in a bimodal FMD (Figure 6a). This bimodality disappears when we consider only spatial groups 1–3, i.e., those with events occurring at close distances to station LTK, which was used for the single-station detections. Station LTK also seems to affect detections in CAT3 as the *M_c_* of 1.4 for the entire CAT3 is reduced to *M_c_* = 0.9 for spatial groups 1–3.

In addition, the numerous detections in CAT2_1–3_ enabled us to explore possible *b*-value temporal variations that may have appeared on the Perachora peninsula during the swarm. The *b*-value expresses the relative numbers of smaller- to larger-magnitude earthquakes and constitutes an integral part of all seismic hazard studies [61,62]. Such temporal variations may reflect changes in the state of stress as the *b*-value is considered inversely proportional to the differential stress [44,45]. CAT2_1–3_ was considered complete down to *M* = 0.0 (Table 1), with a G–R relation holding for almost four orders of magnitude (Figure 7), a wide enough range for robust estimations of the *b*-value [63]. The analysis was performed in temporal windows of 1000 events, sliding every 500 events (50% overlap). Instead of taking a constant *M_c_* = 0.0 for CAT2_1–3_, we considered possible temporal variations and estimated *M_c_* in each temporal window, according to the MaxC method and the GoF test. As was discussed previously, the GoF test for 95% residuals provided more robust *M_c_* values for the G–R relation in CAT2. Hence, it was the one that was adopted in each temporal window. If, however, the GoF test did not return an *M_c_* value for 95% residuals, then we considered the *M_c_* provided by the MaxC method, corrected to +0.2 units of magnitude [64]. In the 1000-event temporal windows, we found that *M_c_* varied from −0.2 to 0.2.

**Figure 8:**
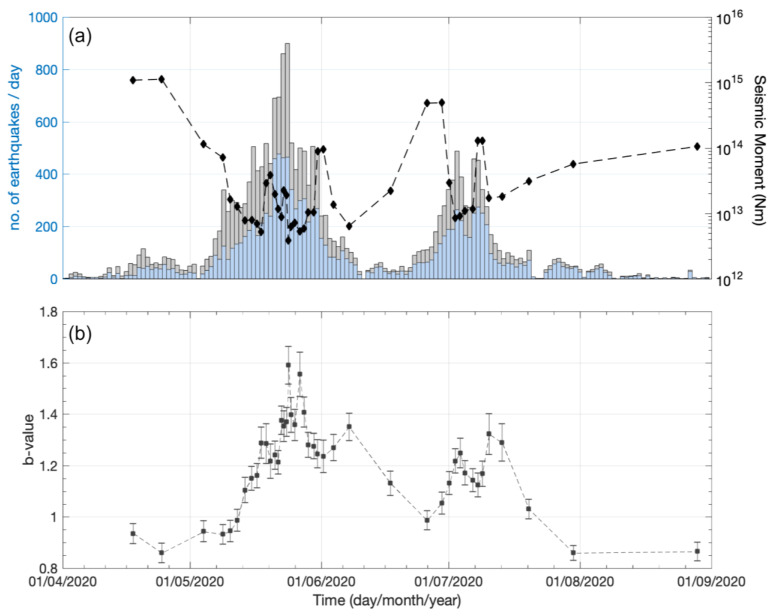
(**a**) Histogram of the number of events per day (gray) and the number of events with *M* ≥ 0.0 (blue) over time (left axis) for CAT2_1-3_. The black diamond symbols, linked by a dashed line, show the cumulative seismic moment release in each temporal window (right axis). (**b**) Temporal variations of the *b*-value (filled squares) and the associated error bars, as estimated from the G–R relation for CAT2_1–3_, in sliding windows of 1000 events with 50% overlap.

The calculated *b*-values and their associated uncertainties as a function of time are presented in Figure 8. The seismicity rate and the cumulative seismic moment release ($\sum M_{o}$) in each temporal window are also plotted. The seismic moment release (in Nm) was approximated with the equation $\log M_{o}=1.5\cdot M+9.1$ [65]. During the period from April to August 2020, when the swarm occurred on the Perachora peninsula, the *b*-value varies considerably within the range of 0.86–1.59. Before the burst of activity in the area, *b*-values are generally around ~0.9. As the swarm activity initiates and the seismicity rate starts to increase during May 2020, *b*-values gradually increase, reaching the highest values during 20–25 May. At the same time, $\sum M_{o}$ reaches the lowest observed values. Following the occurrence of an *M_L_* = 3.2 event on 30 May, the *b*-value decreases to ~1.2 at the end of May, before increasing again to ~1.3 at the beginning of June. At the end of June, the *b*-value decreases to ~1.0 and the $\sum M_{o}$ increases as some moderate-sized events and the largest event (*M_L_* 3.7) of the swarm occur. Following the rise in the seismic activity in the area during late June–early July, the *b*-values increase again to ~1.2–1.3, before gradually decreasing after mid-July to the “background” level of ~0.9. The overall *b*-value variations are in quite good agreement with both the seismicity rate and the seismic moment release, indicating that high *b*-values of 1.2–1.4 are associated with bursts of small-magnitude events, while lower *b*-values of 1.0–1.2 appear with the occurrence of moderate-magnitude events during the swarm.

### 3.2. Spatiotemporal Distribution

**Figure 9:**
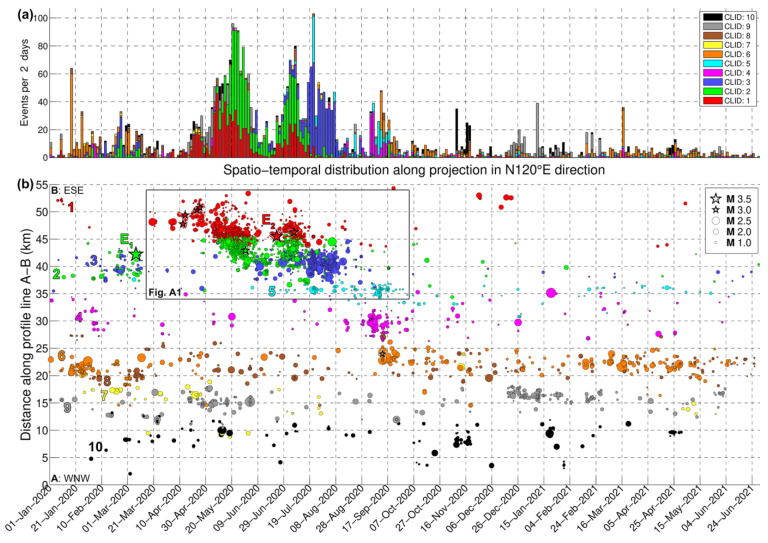
(**a**) Stacked histogram of the number of events per two days for events of CAT3 with magnitude *M* ≥ 0.5. (**b**) Spatiotemporal projection of the epicenters along the WNW–ESE-oriented (N120°E) A–B profile of Figure 3a. Colors and numbers (panel (**b**)) correspond to the spatial groups of Figure 3a (CLID = Cluster ID). Events with magnitude *M* ≥ 3.0 are represented by stars. The major events E_1_ and E_2_ are marked in panel (**b**). The rectangle in panel (**b**) shows the boundaries of the close-up presented in Figure A1 of the Appendix A. Figure A2 of the Appendix A shows the respective cumulative number of events per spatial group.

**Figure A1:**
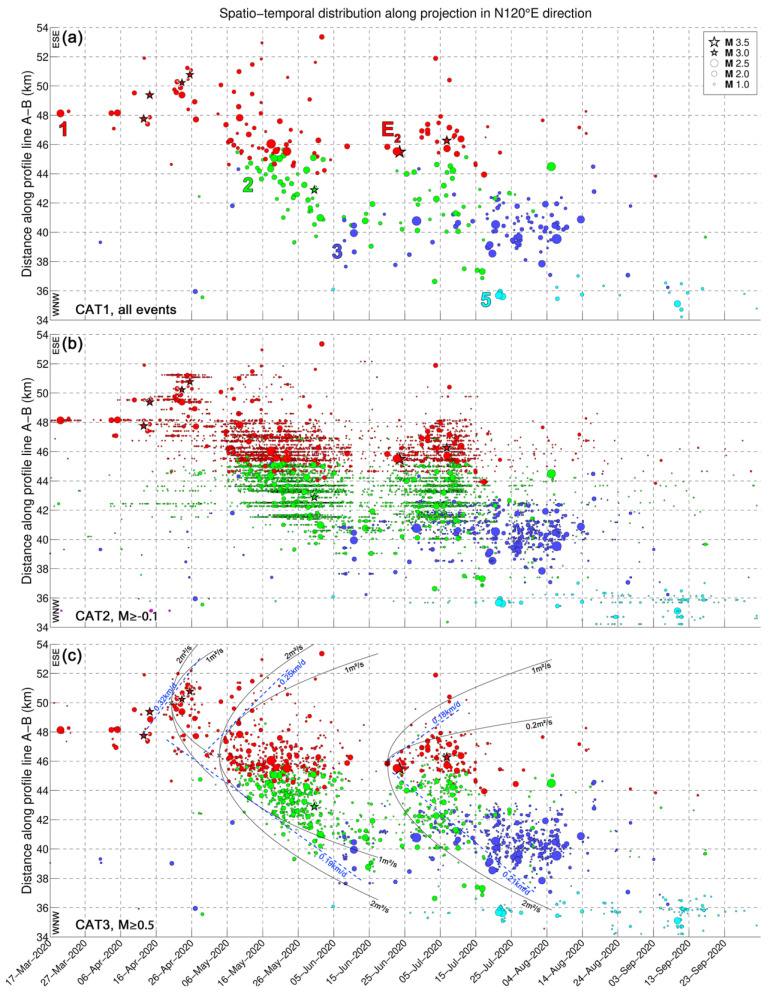
Close-up of the rectangle in Figure 9b, showing the main part of the 2020 Perachora swarm for the three different catalogs: (**a**) catalog CAT1 (templates only) for all events, (**b**) catalog CAT2 (templates and single-channel detections at station LTK with fixed hypocenters to the templates) for events with *M* ≥ −0.1, (**c**) catalog CAT3 (templates and multi-channel detections, relocated) for events with *M* ≥ 0.5. Colors and numbers (panel (**a**)) correspond to the spatial groups of Figure 9, while E_2_ marks the major event of 23 June 2020 in group #1 (red). In panel (**c**), blue dashed lines and labels show the migration speed in km/day along the direction of the projection (N120°E), while black parabolic envelopes indicate triggering fronts of pore pressure diffusion with different values of hydraulic diffusivity, *D*, in m^2^/s.

To assess the enhancement of the catalog in terms of its spatiotemporal distribution, we examined the events of CAT3, which included multi-channel detections when using the matched filter method, after double-difference relocation. As we mostly focused on the evolution of groups 1–3, for this catalog, we considered magnitude thresholds of 0.5, (roughly an order of magnitude below the *M_c_* of CAT1, which contains only the templates) and between 0.1 and 0.9 (the latter being the *M_c_* determined for CAT3_1–3_, i.e., the subset of spatial groups 1–3) for 90% and 95% residuals, respectively. These thresholds filtered out smaller events with very few arrival time data and likely biased relative locations, thus reducing noise in the distribution. In Figure 9b, the epicenters of CAT3 are projected along the WNW–ESE-directed profile line A–B of Figure 3a. The distribution is very similar to that presented by [29] for the initial catalog, containing only the templates, but enhanced in terms of the density of smaller events, filling gaps between the occurrence of larger ones. A comparison of the three catalogs (i.e., CAT1, CAT2, and CAT3) for the main bulk of the Perachora swarm is presented in Figure A1.

**Figure 10:**
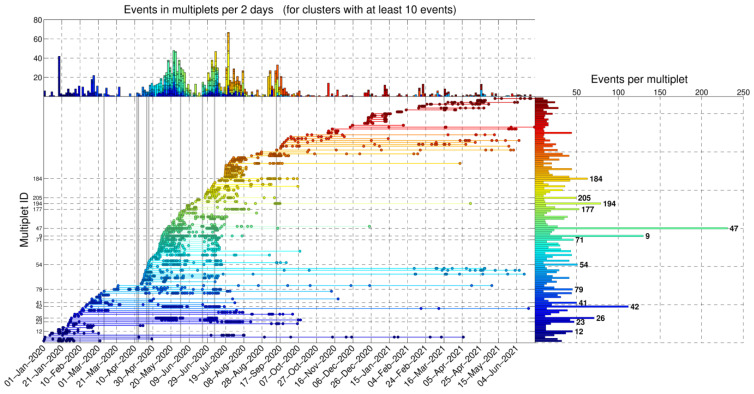
(**Bottom Left**) Multiplet history diagram for events of CAT3 (templates and multi-channel detections) with *M* ≥ 0.5, clustered with farthest-neighbor linkage (at a similarity threshold of 0.5), after removal of multiplets with less than 10 events. Each row is a different multiplet, sorted from bottom to top by ascending order of the origin time of the first event in each cluster. (**Top**) Stacked histogram of the number of events belonging to the selected multiplets every two days, with the horizontal (temporal) axis the same as that of the bottom-left panel. (**Right**) Number of selected events in each multiplet of the corresponding row of the (**Bottom Left**) panel. The occurrence of events with *M* ≥ 3.0 is marked with vertical gray lines in the (**Bottom Left**) panel. The multiplet ID of the largest clusters (containing over 45 events with *M* ≥ 0.5) is marked in the bottom panels.

Herein, we juxtapose the spatiotemporal evolution of the sequence with the history of the generation of new multiplets and the occurrence of repeating events. The latter is presented in Figure 10, which shows a selection of multiplets from CAT3, formed with farthest-neighbor linkage at a threshold of 0.50 (column labeled “far 0.50” in Excel File E3 in the Supplementary Material), with at least 10 events of *M* ≥ 0.5. The largest multiplets are labeled according to their multiplet ID (MID) for identification. Unless described otherwise, MID references in this section refer to cluster numbers in column “far 0.50” of Excel File E3. New multiplets are generated as seismicity activates different structures in the study area. Repeating events may occur in bursts when their multiplet is generated, then pause for a certain time interval and possibly be reactivated later. In some cases, multiplets cease completely as others are created during the progression of the swarm.

**Figure A3:**
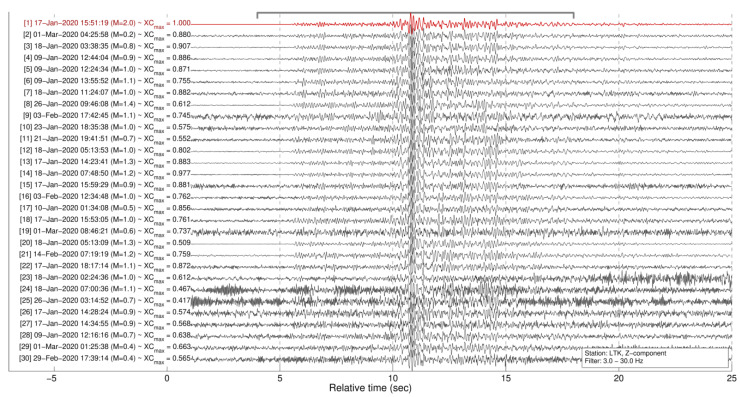
Waveforms of template “2020-01-17-15-51-20” (top, red) and 29 associated detections (black), belonging to multiplet #10 of CAT3, formed with farthest-neighbor linkage at a similarity threshold of 0.50. The recordings are from the vertical component of station LTK, filtered between 3 and 30 Hz. Events of this multiplet are associated with spatial group #6 (orange), about 27 km NW of station LTK, and particularly with a burst of activity on 17–18 January 2020. Note that the presented XC_max_ values are calculated for the purpose of this example and can be regarded as quite low when cross-correlation is performed between noisy records.

**Figure A4:**
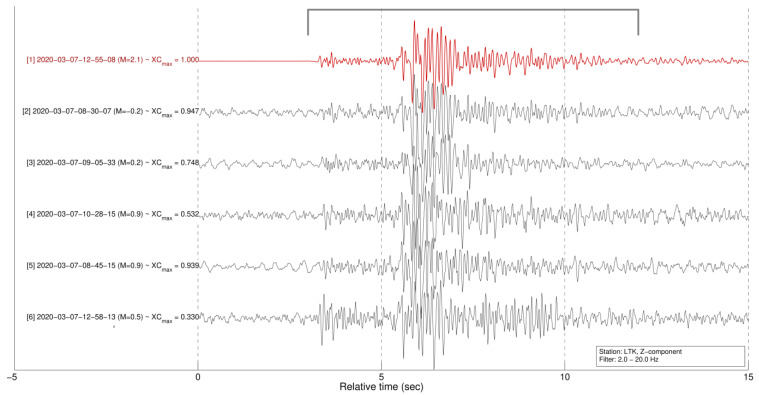
Waveforms of template “2020-03-07-12-55-08” (top, red) and five associated detections (black), belonging to multiplet #114 of CAT2, formed with farthest-neighbor linkage at a threshold of 0.90. The recordings are from the vertical component of station LTK, filtered between 2 and 20 Hz. Specifically, the template is the only aftershock of the *M_w_* = 3.9 event E_1_ in CAT1.

At the beginning of the sequence, more than 60 events occurred in group #6 (orange), about 27 km NW of station LTK, particularly on 17–18 and 26 January 2020. One of the main multiplets in this cluster is MID #10, with some of its recordings at station LTK presented in Figure A3. In February–March 2020, a small excitation was detected in groups #2 and #3, including the occurrence of the major *M_w_* = 3.9 shallow earthquake of 7 March 2020, 08:20 UTC, which apparently did not produce an aftershock sequence, as would be expected for an event of this magnitude. CAT1 contains one aftershock on the same day at 12:55 UTC. It is noted that although multiplets can contain a wide range of magnitudes, waveforms of relatively large events are harder to correlate with smaller ones due to source effects affecting the waveform shape [32,66]. CAT3 contains one event similar to the smaller aftershock; meanwhile, CAT2 contains 12 detections associated with the aftershock. However, several of the former appear to be false detections of a different waveform that only partially resemble the S-waves of the aftershock at the vertical component of station LTK. Some of the valid detections with the aftershock template are presented in Figure A4, all with relative magnitude *M* < 1.0. Two detections were also made of the mainshock, but they were both invalid.

On 17–18 March 2020, a small burst of events occurred in group #1 (red) at a distance of ~48 km along the profile A–B (Figure A1), with one *M* = 2.5 event on 20 March at 01:36 UTC in the initial catalog (CAT1), a few detections in CAT3 (several below *M_c_*), and even more detections in CAT2. This location is related to the origin of the Perachora swarm and multiplet ID 79 of CAT3. The additional detections in CAT2 indicate that this source generated the first events on 17 March. A few more events in group #1 occurred on 3 April in the same area. CAT2 shows a stream of events persisting at that fixed location (Figure A1b). About 30 multiplets with at least 10 events with *M* ≥ 0.5 were generated in the first 3 months. Starting on 16 April, larger events of *M* ≥ 3.0 were triggered and seismicity began migrating ~4 km toward ESE at a rate of ~0.32 km/day (Figure A1c), until about 26 April—or, more likely, a few days after that, as indicated by CAT2 (Figure A1b). A burst of about 17 new multiplets was generated between 16 April and 4 May (Figure 10). From that point on, most of the seismicity occurred at distances < 48 km along profile A–B toward WNW. Until then, CAT1 and CAT3 show almost no activity in group #2 (green). CAT2 indicates that some multiplets of group #2 may have been generated in early April, producing several events.

**Figure A5:**
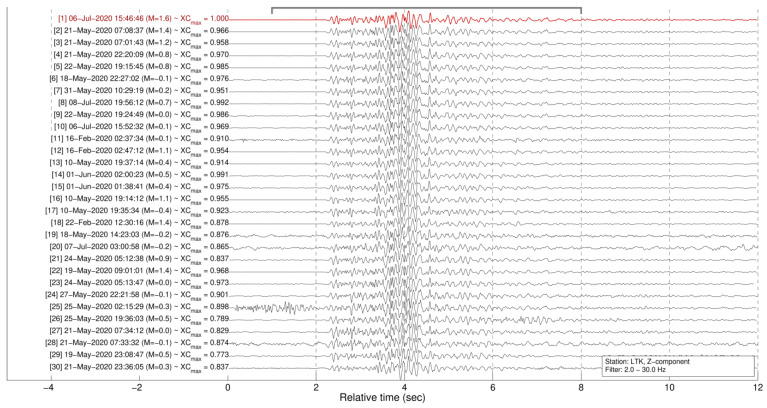
Waveforms of template “2020-07-06-15-46-49” (top, red) and 29 associated detections (black), belonging to multiplet #42 of CAT3, formed with farthest-neighbor linkage at a similarity threshold of 0.50 (see also Figure 10 for the temporal distribution of this multiplet). The recordings are from the vertical component of station LTK, filtered between 2 and 30 Hz.

The first significant increase in the seismicity related to the swarm, involving both groups #1 and #2, began after 6 May, with 10 new multiplets generated in 10 days, including the largest one with MID 47 (Figure 10). Notably, no *M* ≥ 3.0 events occurred during that time, yet the productivity of the swarm was much greater than in the previous days, yielding low cumulative seismic moment release and high *b*-values (Figure 8). In Figure A1c, parabolic envelopes have been drawn, representing the likely triggering fronts caused by pore fluid pressure diffusion, according to the formula by [11]:

(2) $$r\left(t\right)=\sqrt{4\pi Dt},$$

where *r*(*t*) is the time-dependent radius of the triggering front and *D* is the value of hydraulic diffusivity. The positioning and timing of the origin of fluid injection are somewhat arbitrary and guided by the observed spatiotemporal pattern of seismicity, while different values of *D* may apply to distinct episodes of seismicity bursts or migration in a certain direction. Several multiplets, including MIDs 26 and 42 (Figure 10), seem to be re-activated during the first wave of the swarm, which ends on about 4 June. Figure A5 shows examples of recordings matching a certain template of the latter multiplet, indicating that it is activated as early as 16 February, but also during 10 May–1 June, and then again on 6 July. Multiplet ID 26 produces its first events on 31 January. A drop in the seismicity rate and generation of new multiplets is observed between 5 and 22 June, during which group #3 (blue) starts to become activated. CAT2 shows sparse seismicity in group #2 (green) and fewer events in group #1 (red). 

**Figure A6:**
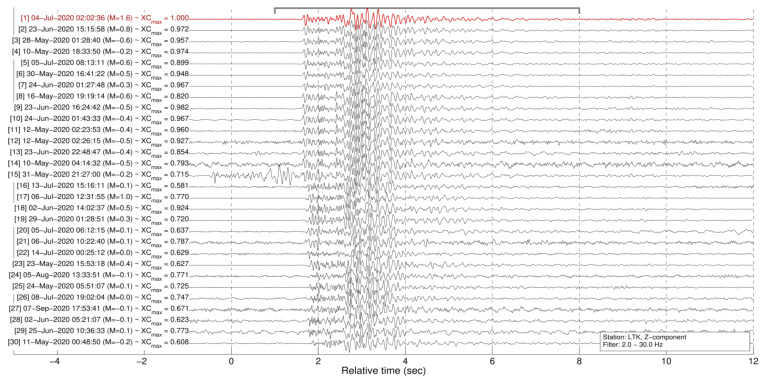
Waveforms of template “2020-07-04-02-02-37” (top, red) and 29 associated detections (black), belonging to multiplet #9 of CAT3, formed with farthest-neighbor linkage at a similarity threshold of 0.50 (see also Figure 10 for the temporal distribution of this multiplet). The recordings are from the vertical component of station LTK, filtered between 2 and 30 Hz. Note how the template matching method manages to detect the overlapping signal within the coda of a previous earthquake in waveform #15.

On 20 and 22 June, a few events occur in group #1, including two with magnitudes 2.1 and 2.6 in CAT1. On 23 June, the *M_w_* = 3.7 event (E_2_ in Figure 3a) occurs in group #1 at Perachora. Interestingly, after E_2_ and up to 01:36 UTC on 29 June, CAT1 contains no events in group #1 and only eight in group #2. However, CAT2 shows that E_2_ triggers a large number of small events in both groups, and CAT3 shows an immediate increase in the seismicity rate. It also triggers events in the multiplet with MID 9, which was first created on 10 May (Figure A6). In Figure A1c, a third series of parabolic triggering fronts has been drawn for this wave of the swarm, starting on 20 June, near E_2_, which is projected at a distance of ~45.5 km along the profile A–B, slightly offset toward WNW relative to the origin of the first *M* ≥ 3.0 events that occurred in April. As was also the case during the first wave, only a few events migrate toward ESE (group #1), at a rate of about 0.18 km/day, and most seismicity spreads at ~0.21 km/day toward WNW. Few events occur in group #3 (blue) during this burst. The seismic activity of this wave stops on 15 July 2020, which is also indicated in CAT2 as very few events occur in groups #1 and #2 after that time. 

**Figure A7:**
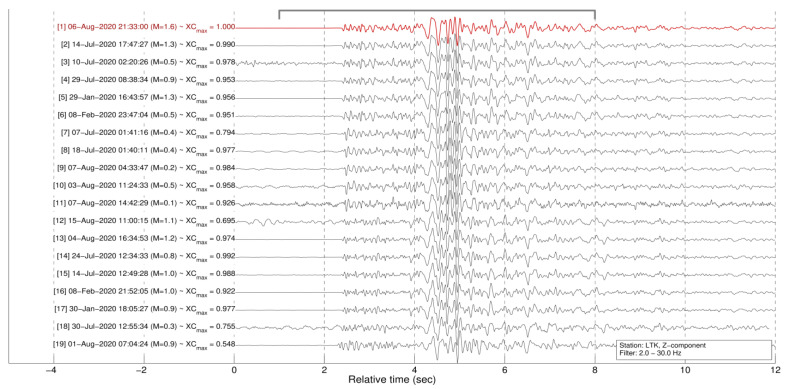
Waveforms of template “2020-08-06-21-33-02” (top, red) and 18 associated detections (black), belonging to multiplet #23 of CAT3, formed with farthest-neighbor linkage at a similarity threshold of 0.50. The recordings are from the vertical component of station LTK, filtered between 2 and 30 Hz. Events of this multiplet are associated with spatial group #3 (blue in Figure 3a), mainly activated after 15 July 2020.

The next wave of this swarm is triggered on 18 July, almost exclusively in group #3 (blue), without any significant earthquake occurring. This generates eight new multiplets with over 10 *M* ≥ 0.5 events in CAT3 on the same day and also reactivates the multiplet with MID 23 (Figure A7) and a few others, which had exhibited activity earlier in 2020. A few events in group #5 (cyan) are also triggered between 21 and 22 July, including two events with *M* = 2.4.

A final burst of events in the Perachora swarm occurs on 3 September, bifurcating toward two different locations, one further offshore WNW in spatial group #4 (purple), and shortly after in group #5, which is toward the SW near Kiato, then group #6 (orange) in the same direction as group #4. The seismicity in this swarm practically ceases by the end of September 2020, with few newly formed multiplets afterward, mainly related to offshore clusters. 

**Figure A8:**
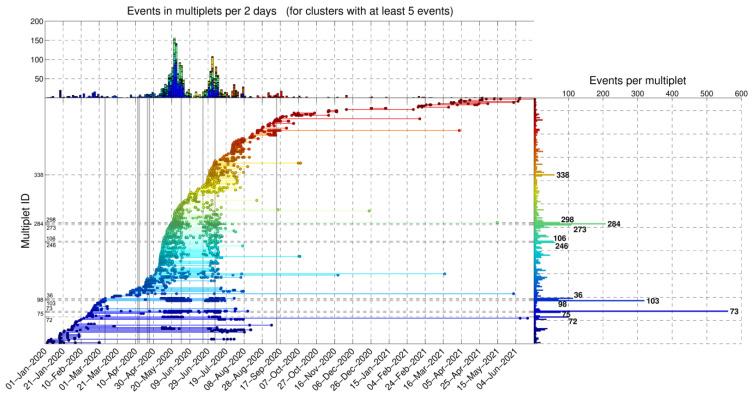
Same as Figure 10, but for CAT2 (templates and single-channel detections on station LTK) with *M* ≥ −0.1, as clustered with farthest-neighbor linkage at a threshold of 0.90 between templates and a correlation value of at least 0.75 between templates and detections, after the removal of multiplets, and with less than five events. The multiplet ID of the largest clusters (containing over 50 events with *M* ≥ −0.1) is marked in the bottom panels.

The multiplet history is displayed in Figure A8 based on a subset of events with *M* ≥ −0.1 in CAT2, with correlation > 0.75 to their respective templates, for multiplets formed with farthest-neighbor linkage at a high threshold of 0.90 and containing at least five events. This selection involves roughly the same number of events as that of CAT3 in Figure 10, but these detections were made with a single channel on station LTK. A prominent difference is the larger number of events in multiplets for the Perachora groups (#1, #2, and #3) and the far fewer events for the more distant offshore clusters. Other than that, the overall pattern is very similar, indicating that even a low-quality catalog of single-channel detections at a local station can provide the main clustering characteristics of a sequence. 

### 3.3. Temporal Properties of Multiplet Families

The high number of events in the enhanced catalogs enables us to study the temporal properties of the multiplet families for the EGoC during the study period. In the analysis, we consider CAT2 as it was derived from template matching on the single-channel detections of station LTK. To ensure homogeneity in the analysis, we only consider the events with relative magnitudes equal to or greater than the magnitude of completeness of CAT2 (i.e., *M_c_* = –0.1), totaling 17,204 events. To group the events in multiplet families, similarity-based clustering is performed with farthest-neighbor linkage, as described in Section 2.2. 

**Figure 11:**
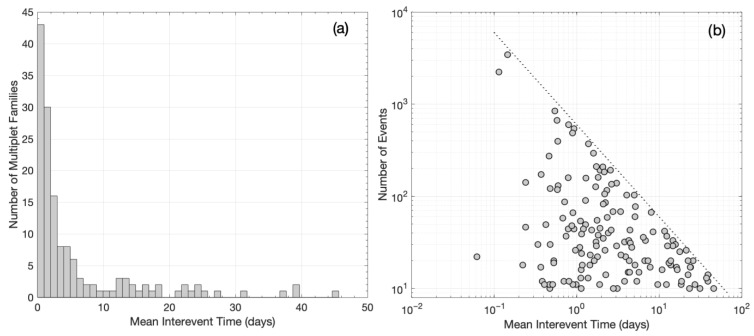
(**a**) Histogram of the mean inter-event times (in days) for the multiplet families with at least 10 events (*N* ≥ 10). (**b**) The number of events in each multiplet family as a function of its mean inter-event time (in days). The dotted line shows an inverse power law function with slope –1.

Initially, for each multiplet family with ten or more detections (*N* ≥ 10), the inter-event times *τ* (or waiting times) between the successive events are calculated as *τ_i_* = *t*_i+1_ − *t_i_*, where *t_i_* is the occurrence time of the *i*th event in each multiplet family, with *i* = 1,2,…,*N*−1 and *N* the total number of detections. In CAT2, there are 148 such multiplet families with *N* ≥ 10, totaling 16,456 events. Then, we estimate the mean inter-event time $\bar{\tau }$ for each multiplet family and construct a histogram of the mean inter-event times (Figure 11a). This analysis shows that most multiplet families in our catalog present mean inter-event times of less than five days (~70%), with the most populous group showing a $\bar{\tau }$ of less than one day (~29%). The multiplet families with short $\bar{\tau }$ are mostly associated with short-term earthquake bursts in spatial groups #1, #2, and #3, which appear during the swarm that occurred at the Perachora peninsula [29]. Furthermore, in Figure 11b, we can see that the two multiplet families with the higher number of detections present quite short $\bar{\tau }$, while a general trend appears with $\bar{\tau }$ increasing as the number of events in each multiplet family decreases. This trend forms an upper bound that scales as an inverse power law with slope –1 (Figure 11b).

**Figure 12:**
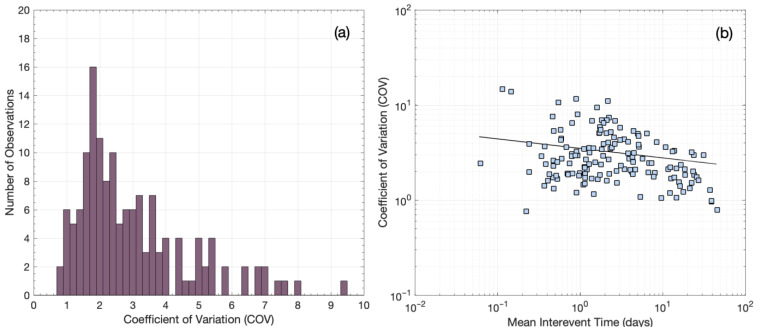
(**a**) Histogram of coefficient of variation (COV) values for the multiplet families with *N* ≥ 10. (**b**) The COV values as a function of the mean inter-event time (in days) for each multiplet family with *N* ≥ 10. The solid line shows an inverse power law function with slope –0.1.

In addition, to investigate clustering or quasi-periodicity in the temporal occurrence of multiplet families, we estimate the coefficient of variation (COV) for each family with *N* ≥ 10. The coefficient of variation (COV) of earthquake inter-event times *τ* is defined as the standard deviation (*σ_τ_*) divided by the mean inter-event time $\bar{\tau }$ [67]. A COV equal to one indicates random Poissonian occurrence, whereas a COV equal to zero, or between zero and unity, indicates periodic or quasi-periodic behavior, respectively. Meanwhile, COV values greater than one show temporal clustering within the multiplet families. In our catalog, the vast majority of multiplet families show COV values greater than unity (Figure 12a), indicating temporal clustering, mostly associated with short-term earthquake bursts. Six families show COV values around unity, which imply Poissonian occurrence, whereas only two families show COV values less than unity, which denote quasi-periodic behavior. The latter result suggests that most multiplet families showing temporal clustering are caused by aseismic factors, such as pore fluid pressure diffusion, which has been identified as the main triggering mechanism of the 2020 Perachora swarm [29]. A constant stressing rate, meanwhile, is associated with random or quasi-periodic behavior [68], which is the case for only a small fraction of multiplet families in our catalog. Furthermore, Figure 12b shows that COV values in our dataset generally decrease toward unity with increasing $\bar{\tau }$, implying that the temporal occurrence of events in longer-term multiplet families tends to occur more randomly.

Temporal clustering in the occurrence of multiplets in each family, as exemplified by the previous result, should also be observed in the probability distribution of inter-event times. To investigate this hypothesis, we estimate the normalized probability density function for the stacked inter-event times of all multiplet families with *N* ≥ 10. Inter-event times *τ* are initially rescaled with the mean inter-event time $\bar{\tau }$ in each family and then stacked together. The probability density is then calculated by counting the number of *τ* falling in logarithmically spaced bins, divided by the bin width and the total number of events, to form the “global” probability density of the rescaled inter-event times *τ* in our catalog. The analysis is then repeated for the families with *N* ≥ 10, revealing correlation coefficients (CC) between the multiplets and their master event equal to or greater than 0.7, 0.8, and 0.9, respectively. 

**Figure 13:**
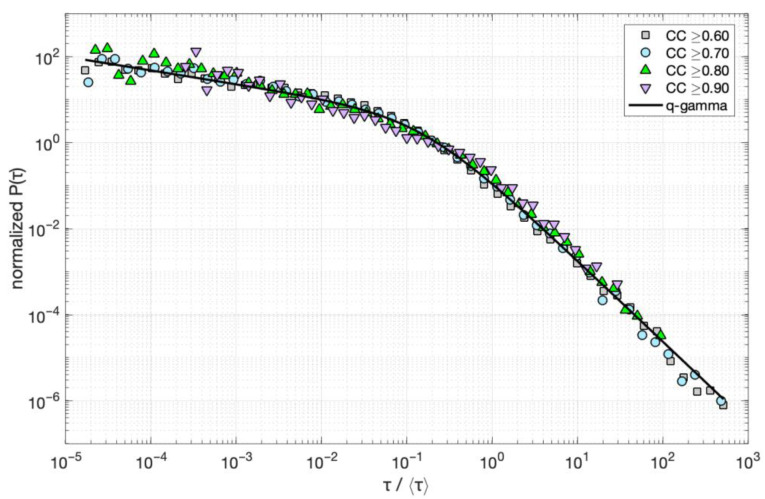
Normalized probability densities *p*(*τ*) of the rescaled inter-event times *τ* (symbols) for the stacked inter-event times of all multiplet families with *N* ≥ 10 and various values of the correlation coefficient (CC) between the multiplets and the master event. The solid line represents the *q*-generalized gamma function (Equation (3)) fitted to the data, with parameter values *C* = 2.5, *γ* = 0.68, *τ*_0_ = 0.1, and *q* = 1.64.

Figure 13 shows the results of this analysis in terms of the normalized probability densities *p*(*τ*) of the rescaled *τ* for the four datasets. Initially, we observe that all four datasets fall into a unique *p*(*τ*) for over eight orders of magnitude, indicating self-similarity in the temporal occurrence of multiplets in each family, regardless of their correlation with the master event. This unique *p*(*τ*) is observed despite the large differences in the size of each dataset, i.e., 16,308 for CC ≥ 0.6; 7043 for CC ≥ 0.7; 2353 for CC ≥ 0.8; 535 for CC ≥ 0.9. For CC ≥ 0.9, greater scattering of the observed values appears due to the small number of events, but it still follows the same trend as the other *p*(*τ*). This trend follows slow power law decay for short $\tau /\bar{\tau }$, i.e., a straight line in the log–log representation of Figure 13, and gradual crossover to faster power law decay for longer $\tau /\bar{\tau }$. We approximate this scaling behavior with the *q*-generalized gamma function [69]:

(3) $$f\left(\tau \right)=C{\left(\frac{\tau }{\tau_{0}}\right)}^{\gamma -1}exp_{q}\left(-\frac{\tau }{\tau_{0}}\right),$$

where *C* is a normalization constant, *τ*_0_ a scaling parameter, *γ* a scaling exponent, and *exp_q_*(*x*) the *q*-exponential function [70,71]:

(4) $$exp_{q}\left(x\right)={\left[1+\left(1-q\right)x\right]}^{1/\left(1-q\right)},$$

In the limit of q→1, the *q*-exponential function recovers the exponential function and the *q*-generalized gamma the ordinary gamma function, respectively, while for *q* > 1, a power law regime appears on the right-hand side of Equation (4). Hence, the *q*-value shows how far, or close, the tail of the distribution is to exponential random behavior, while the *τ*_0_ value marks the crossover point to this second regime. The *q*-generalized gamma function has been found to approximate the inter-event time distributions in nonstationary earthquake time series quite well [69,72,73,74], indicating both short- and long-term clustering effects in the evolution of the earthquake activity. 

As observed in Figure 13, the *q*-generalized gamma function fits rather well with the normalized probability densities *p*(*τ*) for the four datasets. This result indicates that for short *τ*, the observed *p*(*τ*) decreases slowly according to a power law $\sim \tau^{\gamma -1}$, with *γ* = 0.68. For *γ* approaching zero, the decrease is similar to Omori power law decay, characteristic of aftershock sequences. In our case, this decay is much slower, indicating short-term clustering effects in the evolution of burst-like multiplets at breaking asperities that interact with each other. For *τ* higher than a characteristic inter-event time, the observed *p*(*τ*) decreases more rapidly and according to another power law, $\sim \tau^{\left(1-\gamma \right)/\left(1-q\right)}$ [69]. The latter indicates clustering effects in the long-term occurrence of multiplets at breaking asperities that interact with each other, even in longer time scales of days. This result is consistent with temporal clustering, rather than quasi-periodic or random behavior, exemplified by the higher-than-unity COV values found for the vast majority of multiplet families.

## 4. Discussion

In this study, we applied a matched filter technique ([30]) to enhance an earthquake catalog in the Eastern Gulf of Corinth (EGoC) between January 2020 and June 2021, focusing on a pronounced seismic swarm that occurred near Perachora, mainly between March and September 2020. This was an opportunity to examine the utility of this method to provide a large number of previously undetected events associated with the swarm, which improve the depth of analysis of its evolution, as well as explore the benefits and limitations of the method with regard to the quality of the solutions obtained and its effect on the statistics of the enhanced catalogs compared to the original one. Below, we discuss the differences between each catalog and the effects of the addition of events detected by template matching to the general properties of each catalog. We then focus on how the enriched catalogs improve our understanding of the temporal evolution of parameters, such as the *b*-value of the G–R scaling relation.

### 4.1. General Catalog Properties

**Figure A11:**
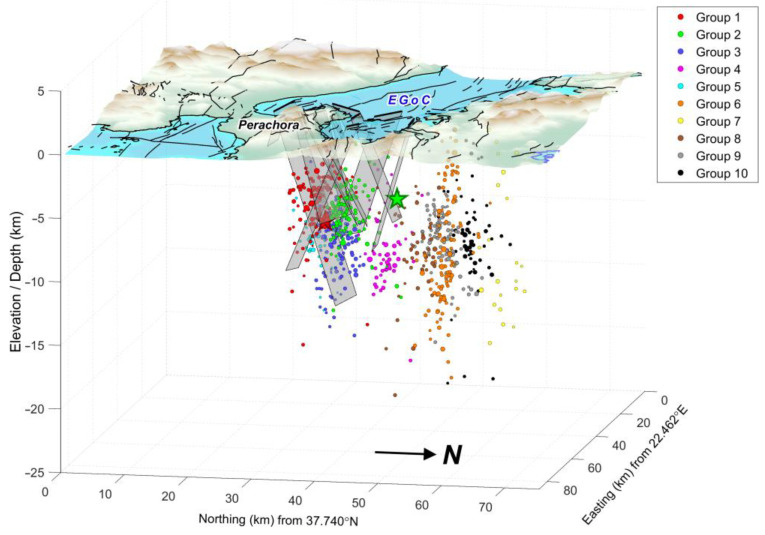
Snapshot of the 3D interactive MATLAB figure available in the Supplementary Materials (File M1). The view is from a low angle toward WSW, with 2× vertical exaggeration. Some of the extruded fault planes at depth are visible, along with the hypocenters of the 10 spatial groups of CAT1 (Figure 3a). Fault lines are from the NOAFaults v4.0 database [48,49].

The EGoC is covered by seismological stations of the HUSN, mainly at its southern flank, near Xylokastro, Kiato, Corinth, and Loutraki, and to the east at Villia (Figure 1). To the north, a station of the HA network (DOMV) has been operational at Domvrena since late July 2021, and there is another of the HP network (DSF) near Desfina to the north-west, though it was not operating in 2020–2021. During the Perachora swarm, the northern side of the EGoC was not covered by local stations. As the gulf is open to the west due to sea cover, this leaves a significant azimuthal gap for earthquakes, which may cause location biases. Furthermore, the lack of a dense local network impedes the detection of low-magnitude events. Nevertheless, the routine catalog CAT1 is considered complete down to magnitude *M_c_* = 1.6 and contains events as small as *M* = 0.6, which can be exploited as templates. The spatial properties of the hypocentral distribution of CAT1 in relation to the known faults in the area were thoroughly analyzed and described by [29]. Here, in addition to the relocated catalog (Excel File E1), we also provide a 3D model for the faults and seismicity in the EGoC (Figure A11) as a 3D interactive MATLAB figure file (File M1 in the supplementary material).

Focusing on one local station (LTK of the HP network, belonging to the HUSN) with data available for the epicentral area of the Perachora swarm, we constructed a template matching catalog (CAT2) with single-channel, full-waveform detections. This beneficially provided the maximum number of detections related to the microseismicity near the station as it could detect signals of very small earthquakes, which other stations further away could not discern, due to the very low signal-to-noise ratio. Indeed, the lowest magnitude in this catalog is −1.4, as determined from the amplitude ratio between detection and the associated template, following the formula of Equation (1). The vast number of initial detections (over 74,000) was reduced to 24,402 by imposing stricter criteria, such as the requirement for a relative magnitude to be calculated, which demands the minimum signal-to-noise ratio. These additional detections—still large in number, even after being reduced—were produced with a minimum amount of processed data, which may well be the only option in some areas of low instrumental coverage, such as the EGoC. Single-station matched filter detections are useful for cases where an area of interest is only covered by one local station with data available, with other stations of the network too far away for the detection of small earthquakes. One such case was the Guy–Greenbrier sequence in central Arkansas, where induced seismicity occurred in an intraplate region with sparse instrumentation. Through single-station template matching, [35] managed to detect 460,000 earthquakes using 1382 templates for the period between July 2010 and October 2011, reducing the magnitude of completeness by 2–3 orders compared to the initial catalog.

However, single-channel detections are prone to false triggers, causing artifacts in both the spatiotemporal distribution of seismicity and the frequency–magnitude distribution (FMD). By assessing the spatiotemporal distribution, detections of lower correlation (associated with specific templates) were removed, as it was observed that they tended to produce false triggers. These templates were not necessarily of low signal-to-noise ratio; some were even associated with the larger events of the sequence. The main issue was their frequency content, which rendered them similar to low-frequency (e.g., pulse-like) local noises, or even signals of events from regional distances. It is noted that this catalog could still contain some false triggers and should be used with caution and after visual confirmation of the signals, though its overall characteristics are generally consistent with the patterns observed in the initial catalog. An improvement, in terms of reliability, could be made by using multi-component templates at the reference station, thus requiring similarity on three channels simultaneously rather than one, but this would increase the system resources and processing time required. Here, we used the “triggers” of the single-channel procedure as a guide to optimize the multi-channel template matching for CAT3 by limiting the search window only around the trigger times of the initial 74,000+ detections, rather than searching the whole length of the continuous records. Another problem with single-station detections is that they can only provide information on the origin time and relative magnitude of the detected events, while the hypocenter is fixed to that of the associated template.

The FMD is further affected by potentially different scaling between the local magnitudes of the initial catalog (CAT1), for the templates, and the relative magnitudes of the detections (CAT2), which may be responsible for the difference in the *b*-value between the smaller and the higher magnitude ranges in the entire CAT2 (Figure 6). Alternatively, this bias may be due to the usage of a single station as, normally, the magnitude is calculated from many stations at different azimuths, covering different parts of an earthquake’s radiation pattern and providing an average. However, the FMD for spatial seismic clusters close to the station LTK, mainly registering the events of the swarm, is continuous down to *M* = 0.0 (Figure 7). This suggests that bimodality in the FMD of the entire CAT2, with different *b*-values for smaller and higher magnitude ranges, is an artifact caused by the under-representation of small-magnitude events in spatial clusters away from LTK, where detectability is low. It further suggests that mixing relative magnitudes with *M_L_* in our case does not seem to affect scaling. 

Some of the above issues with the catalog containing single-channel detections were ameliorated by performing multi-channel matched filter detections (Figure 2). This took advantage of all three components on all operational local stations, with P- and S-waves in separate windows of the vertical and horizontal components, respectively. The requirement for an adequate level of similarity on all channels simultaneously greatly reduces the chance of false alerts and thus produces more reliable results. However, it also limits the capability to detect low-magnitude events recorded only by stations very close to the epicenters. As a result, the FMD in CAT3 presents a different slope in the lower magnitude range. This deficit is mainly attributed to the difficulty of achieving a scenario where enough local stations detect small events. The situation is not surprising as similar differences between initial catalogs and those enhanced by template matching have been previously reported in the literature [60]. For example, [75], who worked on a catalog of 15 years in the Eastern Tennessee Seismic Zone, estimated *b* = 1.48 in the original catalog, with *M_c_* = 2.1, and *b* = 1.16 in the catalog of detections, with *M_c_* = 0.8. The discrete FMD in their template matching catalog has the same issue as the one in Figure 5a for CAT3, with a steeper slope at the higher magnitudes (templates) than the lower ones (mainly detections). CAT2 also presented a bias in the FMD, though to a lesser degree, and this problem became less evident when isolating groups on the Perachora peninsula, near the reference station LTK, showing a continuous distribution down to *M* = 0.0 (Figure 7). This indicates that when examining a more constrained area that is better covered in terms of available data from local stations (or a single reference station, in the case of CAT2), the Gutenberg–Richter scaling relation can be extended down to the lower *M_c_* value of the enhanced catalog without affecting the *b*-value. This has been demonstrated on a large scale in southern California, where [31] detected 1.81 million events using multi-channel template matching of 284,000 templates of events from the SCSN catalog throughout 2000–2017; the magnitude of completeness was reduced from 1.7 to 0.3 while retaining a similar slope in the FMD toward the lower magnitudes (detections).

An additional benefit of the multi-channel matched filter is that it can provide P- and S-wave arrival times, which may be used for single-event location of the detections. This, however, is a feasible approach only when a dense local network is available, capable of providing enough arrival-time picks. In the case of the Perachora swarm, a limited number of phases could be provided with sufficiently acceptable uncertainties. The routine catalog CAT1 was mainly supported by arrival time data for HUSN stations up to an epicentral distance of 200 km [29], but for the matched filter detections (CAT3), stations were only used up to a ~50 km distance. Nonetheless, the obtained P- and S-wave travel times of the detections, in addition to cross-correlation differential travel times, could be used for double-difference relocation of the detections, using the fixed hypocenters of the associated templates as starting locations. With that said, the low number of data, especially for events of small magnitude, could create artifacts in the spatial distribution of the relocated hypocenters, so the results should not be overinterpreted in areas of sparse network coverage. Such biases were more evident for epicenters near Perachora, i.e., close to the local stations, whereas offshore events were less affected. The above observations reflect the trade-off between increasing the number of available events in a catalog and keeping the false alerts and artifacts to a minimum. This needs to be balanced by defining an appropriate threshold value for the similarity metric, i.e., the median absolute deviation or average correlation coefficient, thus ensuring a suitable waveform similarity to retain sufficient detections without creating artifacts, followed by a quality check of the results, including visual inspection and other filters. With EQcorrscan [30], this can be achieved by initially keeping a large number of detections with a low threshold, then inspecting the results, and declustering the catalog by readjusting (increasing) the threshold.

### 4.2. Benefits of the Enhanced Catalogs—Implications for Pore Pressure Diffusion and Stress Changes

The plethora of detections in CAT2 enables a detailed analysis of the *b*-value temporal variations during the occurrence of the swarm. High *b*-values, generally higher than unity, are associated with a larger ratio of smaller- to larger-magnitude events, typically observed in volcanic areas [76] and induced seismicity [77]. High *b*-values can also be attributed to high crustal heterogeneity [78], low stress accumulation [44], or elevated pore pressure, which reduces the effective normal stress [79]. Meanwhile, typical *b*-values in tectonically active areas are around unity [80]. During the Perachora swarm, we can observe significant *b*-value temporal variations (Figure 8b). As the seismicity rate increases in the area, the *b*-value gradually increases from a “background” value of ~0.9 to values > 1.0, reaching ~1.6 during the most prolific stage of the swarm. As the seismicity rate decreases again in the area after ~3 months of intense activity, the *b*-value returns to the “background” value of ~0.9. The good agreement between high seismicity rates and increased *b*-values indicates that these originate from the bulk of the microseismicity during the most productive stages of the swarm. They correspond to periods of low cumulative seismic moment release for windows of a constant number of events with a magnitude above *M_c_* (Figure 8a) as the vast majority of events are of small magnitudes. The most plausible scenario for the high observed *b*-values during the swarm is increased pore fluid pressure in the area. Based on the spatiotemporal evolution of the swarm, [29] suggested that the Perachora swarm was initially triggered by fluid overpressure and was then driven by pore fluid pressure diffusion in a general ESE–WNW direction. In this case, and in an analogy to injection-induced seismicity [81], periods of higher *b*-values correspond to increased pore pressure in the area, in agreement with an inverse relationship between *b*-values and differential stress [44,79,82]. 

Both enhanced catalogs, CAT2 and CAT3, confirm the general pattern of the spatiotemporal evolution of the swarm, concerning the activated area (Figure A1). However, the density of events is increased, filling temporal gaps of CAT1. The new catalogs confirm the mostly unilateral spreading of events toward WNW and reveal no significant microseismic activity before 17 March 2020 or after September 2020 at Perachora. The isolated major event E_1_ is confirmed to have had few aftershocks. The event E_2_ at Perachora, however, triggered a large number of aftershocks, previously unavailable in CAT1. Interestingly, two of the stronger bursts of increase in the seismicity rate, corresponding to distinct waves of the swarm, were not associated with a major event. This observation points to triggering by external forcing, most likely pore pressure diffusion caused by fluid intrusion into the fracture network, as indicated by the observed parabolic front of the spatiotemporal migration. The multiplet history from both CAT2 and CAT3 validates the generation of new clusters, as seismicity spreads to new fractures, but also the reactivation of previously formed multiplets, showing repeated slip on the same fault patches, facilitated by pore pressure transients.

This is further confirmed by the inter-event time analysis of multiplet families. Most families present short mean inter-event times of just a few days, associated with short-term seismic bursts in Perachora during the swarm. They also exhibit high values for the coefficient of variation, indicating temporal clustering likely caused mainly by aseismic factors, such as pore fluid pressure diffusion, and to a lesser extent, by static and dynamic stress transfer effects. This is further exemplified in the “global” probability density of inter-event times *p*(*τ*) of all multiplet families, where at short time scales, *p*(*τ*) decays as a power law with an exponent much lower than unity that characterizes Omori-type aftershock sequences. However, temporal clustering is not restricted only to short time scales but extends to longer ones of several days; *p*(*τ*) for longer *τ* decays faster this time, as another power law (Figure 13). This scaling behavior is well-approximated with the *q*-generalized gamma function, which presents clustering effects at all time scales and memory in the temporal evolution of seismicity [69]. Meanwhile, only a small fraction of multiplets show Poissonian or quasi-periodic behavior that can be associated with a constant stressing rate [68,83]. 

It should be noted that even with the enhanced catalogs, there is no evidence of rapid migration during distinct bursts, unlike, for example, the 2015 Malamata sequence in the Western Gulf of Corinth [16]. In that case, fluid-controlled swarm growth with a slowly expanding front (~125 m/day) was combined with episodes of short-lived spatiotemporal clusters exhibiting a fast migration rate (2.7–10 km/day) over a short distance, attributed to aseismic slip. In the 2020 Perachora swarm, parabolic envelopes fit well in the spatiotemporal distribution of seismicity, albeit asymmetrically, without rapid expansions spreading outside the envelope after major events.

Part of the multi-channel template matching catalog CAT3, presented in this work, has already been used successfully for increasing shear-wave splitting observations in the EGoC [50]. Analysis of the seismic anisotropy of shear-wave arrivals of crustal earthquakes is a demanding process that requires the application of strict criteria that create a lot of rejected data. An initial catalog of 991 events was enhanced with 4908 additional detections from CAT3, with clear recordings at station LTK. The final dataset of splitting observations comprised 1357 measurements (most of them from arrivals determined by template matching). As ~23% of shear-wave arrivals yielded quality results (with such a low percentage common in shear-wave splitting studies), the exclusive use of events from CAT1 would lead to a very low number of observations. The increased data volume obtained from the template matching arrivals of CAT3 permitted a detailed analysis of splitting characteristics and revealed changes in the rock volume, possibly as a response to stress changes associated with fluid migration along pressure gradients.

## 5. Conclusions

The application of matched filter detection to the January 2020–June 2021 seismicity of the EGoC, particularly for the earthquake swarm at Perachora that evolved between March and September 2020, has highlighted a plethora of events previously missing from routine catalogs. Template matching was applied in two different ways: (1) using full waveforms (P and S) in a single channel of one reference station located within the epicentral area of the swarm (CAT2), and (2) using all the available local stations, with separate P- and S-wave windows for different channels (CAT3). By applying this procedure, we managed to reduce the magnitude of completeness of the catalog down to *M* = 0.0 in the epicentral area of the swarm. The results confirm the main waves of the swarm’s evolution and also emphasize their intensity in terms of the produced seismicity. Furthermore, they highlight a significant increase in the seismicity rate triggered by external forcing, probably a pore pressure transient, without stress transfer due to a major earthquake. A pore pressure triggering mechanism likely produces the high *b*-values that are observed during the swarm and the clustering effects at all time scales in the temporal evolution of the multiplet families. In addition, the enhanced catalogs confirm the lack of aftershocks produced by the major *M_w_* = 3.9 event of 7 March 2020, ~10 km north of the Perachora swarm. 

However, we also note some anomalies in the frequency–magnitude distribution (FMD) of the enhanced catalogs and issues caused by the poor network coverage in the study area. The single-station enhanced catalog (CAT2) presents a bimodal FMD, which may be explained by its poor capability to detect smaller events at the offshore clusters. This bimodality disappears when considering only the spatial groups on the Perachora peninsula. Furthermore, the lack of a dense local network in the area limits the capabilities of the double-difference relocation performed on CAT3 as low-magnitude events with very few data are prone to location biases. Although the location quality of the additional events is inferior to that of those derived from manual analysis, such events are important for research that requires a large data sample, such as that aimed at charting the temporal evolution of shear-wave splitting parameters [50], or the herein presented research into the temporal evolution of the *b*-value of the Gutenberg–Richter scaling relation, associated with variations in the level of stress. The enhanced catalogs produced in the present work should be useful to support future studies of the spatiotemporal properties of microseismicity in the EGoC area associated with aseismic stresses such as fluid overpressure.

## Figures and Tables

**Table 1:** Information on events contained in the three catalogs produced in this study. CATX_1–3_ refers to the catalog for spatial groups 1–3. *M*_min_ denotes the minimum magnitude and *M_c_* the magnitude of completeness, as determined by the GoF test for 95% residuals.

Catalog	Description	*M*_min_	*M_c_*	Total Events	Events with *M* ≥ *M_c_*
CAT1	Templates (relocated)	0.6	1.6	879	458
CAT1_1–3_	>> (groups 1–3)	0.6	1.6	356	205
CAT2	Templates + single-station (LTK)/single-channel (Z) detections, fixed to template hypocenters	−1.4	−0.1	24,402	17,204
CAT2_1–3_	>> (groups 1–3)	−1.4	0.0	22,373	12,861
CAT3	Templates + multi-channel detections, relocated	−0.8	1.4	8527 *	4294
CAT3_1–3_	>> (groups 1–3)	−0.8	0.9	6128 **	1147

* There are 8527 events with the magnitude available and 11,638 in total; ** with only the magnitude available.

**Figure A2:**
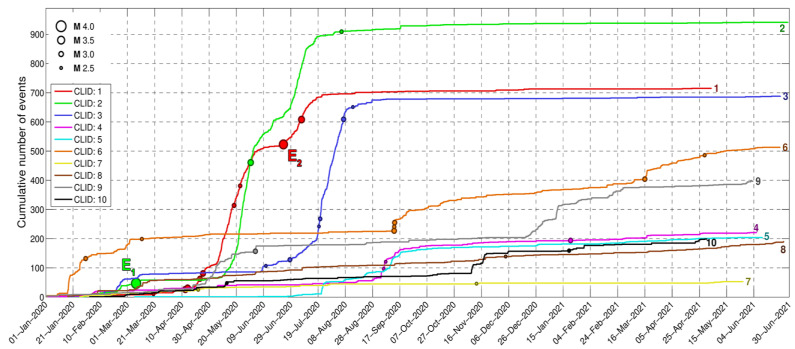
Cumulative number of events per spatial group, represented by different colors and numbers, for a subset of CAT3 containing events with *M* ≥ 0.5. Circles denote events with *M* ≥ 2.5. The major events E_1_ (7 March 2020, *M_w_* = 3.9, in group #2) and E_2_ (23 June 2020, *M_w_* = 3.7, in group #1) are marked.

**Figure A9:**
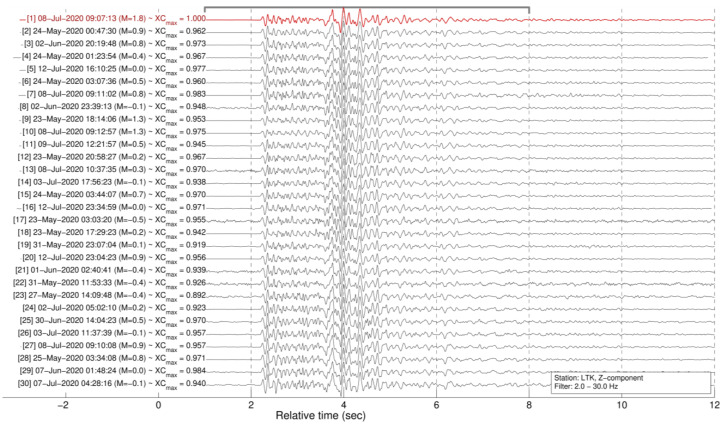
Waveforms of template “2020-07-08-09-07-13” (top, red) and 29 associated detections (black), belonging to multiplet #47 of CAT3 (multi-channel detections), formed with farthest-neighbor linkage at a similarity threshold of 0.50 (see also Figure 10 for the temporal distribution of this multiplet). The recordings are from the vertical component of station LTK, filtered between 2 and 30 Hz. The waveform amplitudes are normalized within the displayed window. XC_max_ denotes the cross-correlation maximum between each waveform and the one directly above in the window marked with a gray bracket at the top. Note the high degree of similarity (XC_max_ > 0.9) between consecutive pairs.

**Figure A10:**
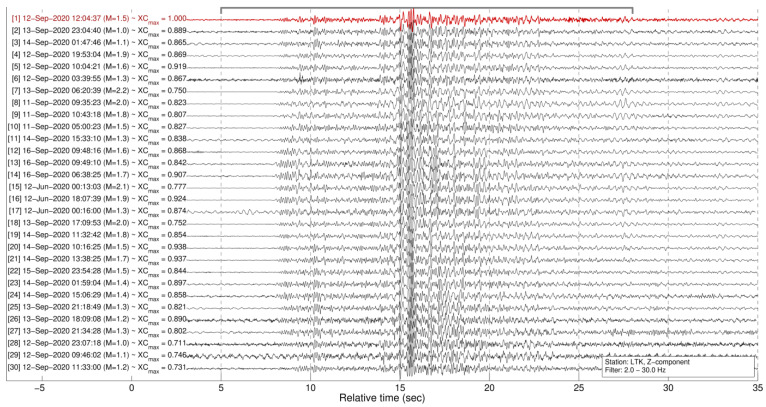
Waveforms of template “2020-09-12-12-04-30” (top, red) and 29 associated detections (black), belonging to multiplet #194 of CAT3, formed with farthest-neighbor linkage at a similarity threshold of 0.50 (see also Figure 10 for the temporal distribution of this multiplet). The recordings are from the vertical component of station LTK, filtered between 2 and 30 Hz.

## Data Availability

Initial catalog data are from the databases of the Seismological Laboratory of the National and Kapodistrian University of Athens (NKUA-SL; http://www.geophysics.geol.uoa.gr/stations/gmapv3_db/index.php?lang=en, accessed on 28 February 2023) and the Geodynamics Institute of the National Observatory of Athens (GI-NOA; http://bbnet.gein.noa.gr/HL/databases/database, accessed on 28 February 2023). The focal mechanisms of Figure 1 are from the databases of the NKUA-SL, GI-NOA, and the International Seismological Centre (ISC; http://www.isc.ac.uk/iscbulletin/search/fmechanisms/, accessed on 28 February 2023). For the application of the template matching method, waveform data from stations of HA [46] and HP [47] networks were acquired from the European Integrated Data Archive (EIDA) node, hosted at the GI-NOA ([52]; http://eida.gein.noa.gr/webdc3/, accessed on 28 February 2023). The three final seismicity catalogs (CAT1, CAT2, CAT3) produced in this study are available in the Supplementary Materials.

## Supplementary Materials

The following supporting information can be downloaded at: https://www.mdpi.com/article/10.3390/s23062923/s1, Excel File E1: Catalog of routine seismicity (CAT1). Seismicity catalog of the Eastern Gulf of Corinth for the period January 2020–June 2021; Excel File E2: Catalog of templates and single-channel detections (CAT2). Seismicity catalog of the Eastern Gulf of Corinth for the period January 2020–June 2021, including matched filter detections using the vertical component of the reference station LTK. The hypocenters of the detections are fixed to the hypocenter of the associated templates; Excel File E3: Catalog of templates and multi-channel detections (CAT3). Seismicity catalog of the Eastern Gulf of Corinth for the period January 2020–June 2021, including multi-channel matched filter detections using the available local stations. The hypocenters of the detections are either fixed to the hypocenter of the associated templates or relocated using the arrival-time picks acquired from the template matching procedure; File M1: Interactive 3D MATLAB figure file. Interactive 3D visualization of the Eastern Gulf of Corinth and the relocated seismicity of CAT1 (January 2020–June 2021). The figure includes a digital elevation model (DEM), fault traces (from the NOAFaults v4.0 database [48,49]), fault planes at depth, and hypocenters of seismicity (CAT1) with colors corresponding to the 10 spatial groups (Figure 3a). The user can view or hide different layers through the available “Layers” menu at the top, or select different viewing angles, vertical exaggeration, and opacity of the DEM through the “3D View” menu. Earthquakes with *M* ≥ 3.5 are depicted as stars. The figure is compatible with MATLAB versions R2009b or above.
