# Supplementary material for: Cluster Analysis of Seismicity in the Eastern Gulf of Corinth Based on a Waveform Template Matching Catalog
Source: Sensors (Basel). 2023 Mar 8;23(6):2923. doi: 10.3390/s23062923 (PMC10056727; doi:10.3390/s23062923)
Supplement: Supplementary file 1 [file sensors-23-02923-s001.zip › Supplementary Material.pdf]

Supplementary Material

for

Cluster Analysis of Seismicity in the Eastern Gulf of Corinth Based on a Waveform Template Matching Catalog

Vasilis Kapetanidis[1], Georgios Michas[1], Ioannis Spingos[1], George Kaviris[1,*] and Filippos Vallianatos[1,2]

[1] Section of Geophysics-Geothermics, Department of Geology and Geoenvironment, School of Sciences, National and Kapodistrian University of Athens, Panepistimiopolis Zografou, 15784 Athens, Greece; vkapetan@geol.uoa.gr (V.K.); gemichas@geol.uoa.gr (G.M.); ispingos@geol.uoa.gr (I.S); gkaviris@geol.uoa.gr (G.K.); fvallian@geol.uoa.gr (F.V.)

[2] Institute of Physics of Earth's Interior and Geohazards, UNESCO Chair on Solid Earth Physics and Geohazards Risk Reduction, Hellenic Mediterranean University Research Center, 71410 Heraklion, Greece

[*] Correspondence: gkaviris@geol.uoa.gr; Tel.: +30-210-7274841

Table of Contents

Excel File E1: Catalog of routine seismicity (CAT1) 2
Excel File E2: Catalog of templates and single-channel detections (CAT2) 3
Excel File E3: Catalog of templates and multi-channel detections (CAT3) 4
File M1: 3D Interactive MATLAB figure file 5
References 6

Excel File E1: Catalog of routine seismicity (CAT1)

Excel file “E1_CAT1_perachora_templates-only.xlsx”.

Seismicity catalog of the Eastern Gulf of Corinth for the period January 2020 – June 2021.

<u>Column description:</u>

1-6) Year, Month, Day, Hour, Minute, Second of origin time.
7) Latitude (°N).
8) Longitude (°E).
9) Focal Depth (km).
10) Local magnitude (M_L).
11) Spatial ID: spatial group number, where available (Figure 3a).
12) MinDist: Minimum distance of the closest station with available data
13) RMS: Root mean square of travel-time residuals (sec).
14) Npha: Number of P and S phases.
15) Gap: Azimuthal gap (°).
16) Nsta: Number of stations with available data.
17) event-code: characteristic unique code label for each event in a yyyy-mm-dd-HH-MM-SS format.
18) Near C0.64: ID numbers of multiplets formed with the nearest neighbor linkage at a threshold of 0.64.
19) Far C0.50: ID numbers of multiplets formed with the farthest linkage at a threshold of 0.50.
20) Description: “Template loc”, for absolute locations provided by VELEST [1], “Template reloc” for relocated solutions with HypoDD.

Excel File E2: Catalog of templates and single-channel detections (CAT2)

Excel file “E2_CAT2_perachora_LTK-detections_fixed-locations.xlsx”.

Seismicity catalog of the Eastern Gulf of Corinth for the period January 2020 – June 2021, including matched filter detections using the vertical component of the reference station LTK. The hypocenters of the detections are fixed to the hypocenter of the associated templates. Multiplet and spatial cluster IDs for the detections are assigned according to the respective cluster to which their associated template belongs. Multiplet IDs are sorted by ascending order of the origin time of the first event in each cluster (including both templates and detections).

Column description:

1-6) Year, Month, Day, Hour, Minute, Second of origin time.

7) Latitude (°N).

8) Longitude (°E).

9) Focal Depth (km).

10) Local magnitude (M_L) for templates, relative magnitude for detections (using Eq. 1).

11) Type: “1” for templates, “2” for detections. Rows corresponding to templates are also highlighted with light green color.

12) Event-code: characteristic unique code label for each event in a yyyy-mm-dd-HH-MM-SS format.

13) Template code: characteristic unique code label in a yyyy-mm-dd-HH-MM-SS format for the template associated with each detection. In case of a template, the code is the same as in column 12.

14) Near C0.64: ID numbers of multiplets formed with the nearest neighbor linkage at a threshold of 0.64 on the correlation matrix of the templates.

15) Spatial ID: spatial group number, where available (Figure 3a).

16) Far C0.50: ID numbers of multiplets formed with the farthest linkage at a threshold of 0.50 on the correlation matrix of the templates.

17) Far C0.60: ID numbers of multiplets formed with the farthest linkage at a threshold of 0.60 on the correlation matrix of the templates.

18) Far C0.70: ID numbers of multiplets formed with the farthest linkage at a threshold of 0.70 on the correlation matrix of the templates.

19) Far C0.80: ID numbers of multiplets formed with the farthest linkage at a threshold of 0.80 on the correlation matrix of the templates.

20) Far C0.90: ID numbers of multiplets formed with the farthest linkage at a threshold of 0.90 on the correlation matrix of the templates.

21) Description: “Template loc”, absolute location for a template provided by VELEST [1], “Template reloc” relocated solutions for a template with HypoDD, “Detection fixed loc” location for a detection fixed to the initial hypocenter of the associated template, “Detection fixed reloc” location for a detection fixed to the relocated hypocenter of the associated template.

22) avg. Corr. Coef.: Correlation coefficient between the detection and the associated template on the vertical channel of station LTK.

Excel File E3: Catalog of templates and multi-channel detections (CAT3)

Excel file “E3_CAT3_perachora_templates+detections_relocated.xlsx”.

Seismicity catalog of the Eastern Gulf of Corinth for the period January 2020 – June 2021, including multi-channel matched filter detections using the available local stations. The hypocenters of the detections are either fixed to the hypocenter of the associated templates or relocated using the arrival-time picks acquired from the template matching procedure. Multiplet and spatial cluster IDs for the detections are assigned according to the respective cluster to which their associated template belongs. Multiplet IDs are sorted by ascending order of the origin time of the first event in each cluster (including both templates and detections).

Column description:

1-6) Year, Month, Day, Hour, Minute, Second of origin time.

7) Latitude (°N).

8) Longitude (°E).

9) Focal Depth (km).

10) Local magnitude (M_L) for templates, relative magnitude for detections (using Eq. 1).

11) Type: “1” for templates, “2” for detections. Rows corresponding to templates are also highlighted with light green color.

12) Event-code: characteristic unique code label for each event in a yyyy-mm-dd-HH-MM-SS format.

13) Template code: characteristic unique code label in a yyyy-mm-dd-HH-MM-SS format for the template associated with each detection. In case of a template, the code is the same as in column 12.

14) Near C0.64: ID numbers of multiplets formed with the nearest neighbor linkage at a threshold of 0.64 on the correlation matrix of the templates.

15) Spatial ID: spatial group number, where available (Figure 3a).

16) Far C0.50: ID numbers of multiplets formed with the farthest linkage at a threshold of 0.50 on the correlation matrix of the templates.

21) Description: “Template loc”, absolute location for a template provided by VELEST [1], “Template reloc” relocated solutions for a template with HypoDD, “Detection fixed loc” location for a detection fixed to the initial hypocenter of the associated template, “Detection fixed reloc” location for a detection fixed to the relocated hypocenter of the associated template, “Detection relocated” relocated hypocentre of a detection based on the picks acquired from the template matching procedure.

File M1: 3D Interactive MATLAB figure file

File "M1_EGoC_3D_model.fig".

Interactive 3D visualization of the Eastern Gulf of Corinth and the relocated seismicity of CAT1 (January 2020 – June 2021). The figure includes a digital elevation model (DEM), fault traces (from the NOAFaults v4.0 database [2,3]), fault planes at depth, and hypocenters of seismicity (CAT1) with colors corresponding to the 10 spatial groups (Figure 3a). The user can view or hide different layers through the available "Layers" menu at the top, or select different viewing angles, vertical exaggeration and opacity of the DEM through the "3D View" menu. Earthquakes with M≥3.5 depicted as stars. The figure is compatible with MATLAB versions R2009b or above.

References

[1] Michas, G.; Kapetanidis, V.; Spingos, I.; Kaviris, G.; Vallianatos, F. The 2020 Perachora Peninsula Earthquake Sequence (East Corinth Rift, Greece): Spatiotemporal Evolution and Implications for the Triggering Mechanism. *Acta Geophys.* **2022**, *70*, 2581–2601, doi:10.1007/s11600-022-00864-x.

[2] Ganas, A.; Oikonomou, I.A.; Tsimi, C. NOAfaults: A Digital Database for Active Faults in Greece. *Bull. Geol. Soc. Greece* **2013**, *47*, 518, doi:10.12681/bgsg.11079.

[3] Ganas, A. NOAFAULTS KMZ Layer Version 4.0 (V4.0) [Data Set]. *Zenodo* **2022**, doi:10.5281/zenodo.6326260.
